# Testing of Pre‐Operative Peripheral Nerve Blocks in Randomised Controlled Trials: A Scoping Review

**DOI:** 10.1111/aas.70211

**Published:** 2026-02-26

**Authors:** Aurelien‐Xuan Rosendal Bahuet, Rasmus Linnebjerg Knudsen, Mathias Therkel Steensbæk, Sina Yousef, Rikke Helene Frølund Bjulf, Anne‐Sofie Linde Jellestad, Jonas Erasmus Egede Glahn, Sine Wichmann, Kai Henrik Wiborg Lange, Lars Hyldborg Lundstrøm, Anders Kehlet Nørskov

**Affiliations:** ^1^ Department of Anaesthesiology Copenhagen University Hospital Hillerød Denmark; ^2^ Department of Clinical Medicine University of Copenhagen Copenhagen Denmark

## Abstract

**Background:**

Peripheral nerve blocks are widely used for anaesthesia in upper and lower limb surgery, but the methods used to assess their success vary substantially. This scoping review examined contemporary research practises and the extent to which trials report on peripheral nerve block evaluation.

**Methods:**

A search was conducted on PubMed for randomised controlled trials published between 2014 and 2025 in anaesthesia journals, involving pre‐operative peripheral nerve blocks in adults undergoing limb surgery. Two independent authors screened and extracted data for each trial. Outcomes included the proportion of trials that reported block testing, described test methods, defined successful blocks, and reported success rates.

**Results:**

Of 284 trials included, 215 (76%, 95% CI 71%–81%) reported testing blocks, and 210 (74%, 95% CI 69%–79%) described the test methods. Of the 215 trials reporting testing, 204 used sensory assessments (95%, 95% CI 91%–97%) and 157 used motor assessments (73%, 95% CI 67%–79%). Success criteria were defined in 164/284 trials (58%, 95% CI 52%–63%), and the same number reported success rates. In the 164 trials reporting on peripheral nerve block success, the median success rate was 98% (IQR 92%–100%).

**Conclusion:**

This scoping review investigated contemporary practise in test methodology reporting in RCTs involving PNBs. We found that although most trials do report testing PNBs, about one in four did not. Furthermore, approximately two in five trials did not define what constituted a successful block or report PNB success rates. Lack of consistent and transparent test methodology poses challenges when comparing trials and performing meta‐analyses, and in translating trial findings into clinical practise. This scoping review exposes a methodological blind spot in regional anaesthesia research. Despite widespread use of peripheral nerve blocks, a substantial proportion of randomised trials fail to report testing methods, success definitions, or handling of failed blocks. Such omissions undermine interpretability, comparability, and clinical translation, and strengthen the case for standardised reporting of regional anaesthesia assessment in future trials.

## 1 Introduction

Heterogeneity in outcome measurement is a commonly cited issue when performing systematic reviews [1]. When testing procedural interventions in randomised clinical trials (RCTs), it is imperative to ensure that the procedure is conducted and performed according to a set standard. Peripheral nerve blocks (PNBs), in which local anaesthetics are injected perineurally to anaesthetise a target area, are an increasingly commonly used anaesthetic procedural intervention [2]. Numerous clinical trials have investigated the effects of different local anaesthetics and doses, as well as adjuvants, in various PNBs [3, 4, 5, 6]. However, to ensure the validity of results, it is crucial that the blocks are systematically and preferably uniformly tested; success rates should also be reported and discussed, with unsuccessful procedures being accounted for when interpreting results [7]. Success rates of PNBs vary according to factors such as needle guidance technique, block type, operator experience, the type of local anaesthetic and the concentration, dose, and volume of local anaesthetic used; a Cochrane systematic review comparing ultrasound guidance to other methods of nerve localisation for PNBs reported block success rates ranging from 61% to 100% [8].

The success of a PNB can be defined in several ways, and previous research suggests there is significant variability in the way PNBs are tested, both in clinical practise and research [9]. Simple sensory and motor function assessments using graded scales remain the most used modalities, but other methods such as thermography or peripheral flow index may also be employed [9, 10]. Variations in testing methodology, such as the time of testing or the grading scale used, may complicate comparisons even between trials assessing PNBs using the same modality [11].

As the use of PNBs continues to increase, so too does the need for a robust and transparent evidence base that can inform clinical practise. Detailed descriptions of how PNBs are tested and deemed successful and the rates of successful blocks are therefore essential to allow valid comparisons between trials, to perform meta‐analysis of results and for clinical applicability of results. To our knowledge, no evidence exists on whether RCTs transparently describe the testing methods, definitions of success and success rates of PNBs.

## 2 Aim

This scoping review was carried out to explore existing literature and assess contemporary research practise in the field of peripheral nerve blocks [12]. It aimed to determine the proportion of RCTs published in the last decade involving PNBs in the pre‐operative anaesthetic management of upper and lower limb surgery that describe how PNBs are tested. We also aimed to explore the proportion of RCTs that had defined a successful block and that reported success rates of blocks.

## 3 Materials and Methods

A protocol was developed in accordance with Joanna Briggs Institute (JBI) guidance [13]. It was made available on Open Science Framework prior to data collection and was published in April 2025 [14, 15]. The scoping review follows the JBI Manual for Evidence Synthesis and adheres to the Preferred Reporting Items for Systematic reviews and Meta‐Analyses extension for scoping reviews (PRISMA‐ScR) [12, 16].

### 3.1 Eligibility Criteria

*Population*

- Studies on adults (as defined by trialists) receiving a pre‐operative PNB as part of, or as the sole, anaesthetic management for upper or lower limb surgery. Truncal and fascial plane blocks were not included, as the lack of consensus on how these blocks can be assessed would have limited the meaningful assessment of this study's outcomes [17, 18, 19].

*Concept*

- PNBs of isolated nerve structures or nerve plexuses with an innervation area accessible for specific, non‐invasive testing of success. If combined with other forms of anaesthesia that would make testing unfeasible (e.g., spinal or general anaesthesia), the PNB was to be performed prior to this.

*Context*

- PubMed‐indexed RCTs published in peer‐reviewed journals of anaesthesia.
- Publication date between January 1st, 2014, and February 20th, 2025.
- Published in English.

### 3.2 Outcomes

- The proportion of RCTs stating that PNBs were tested.
- The proportion of RCTs describing how the PNBs were tested.
- The proportion of RCTs using sensory function to test PNBs.
- The proportion of RCTs using motor function to test PNBs.
- The proportion of RCTs defining PNB success criteria.
- The proportion of RCTs reporting PNB success rates.
- The success rate of PNBs is reported by trialists.
- The proportion of RCTs describing how data from unsuccessful PNBs were managed in the statistical analyses.

### 3.3 Information Sources and Search Strategy

In order to include specialty‐relevant trials with high quality methodology that would accurately answer the review's aims, the search was narrowed to journals listed under the ‘Anesthesiology’ category on Web of Science that primarily focus on perioperative or regional anaesthesia and publish in English [20]. This was determined using the journals' overview, aims and scope, and by screening publications from the past 24 months. A search string adapted to the PubMed database was developed with a medical librarian experienced in database literature searches. As all included journals were indexed on PubMed, the search was not carried out on other databases. The journals included in the search are listed in Table 1, and the search string is provided in Appendix A. The final search was executed on February 20th, 2025.

**TABLE 1:** List of selected journals.

Anesthesiology
British Journal of Anaesthesia
Anaesthesia
Regional Anaesthesia and Pain Medicine
Journal of Clinical Anaesthesia
Best Practise & Research Clinical Anaesthesiology
Anaesthesia & Analgesia
European Journal of Anaesthesiology
Korean Journal of Anesthesiology
Anaesthesia Critical Care & Pain Medicine
Canadian Journal of Anaesthesia
Minerva Anestesiologica
Journal of Anaesthesia
BMC Anesthesiology
Current Opinion in Anesthesiology
Journal of Clinical Monitoring and Computing
Acta Anaesthesiologica Scandinavica
Anaesthesia and Intensive Care

### 3.4 Source Selection and Data Charting Process

Each trial was independently screened by two different authors. Disagreements were resolved through discussion between the screening authors and, where necessary, the involvement of a third, senior author. Data extraction was also performed independently by two authors per trial, with consensus provided by the review's first author. Covidence, a systematic review software, was used for trial screening and data extraction [21].

### 3.5 Data Items

Data for each trial were collected regarding trial information (e.g., year of publication, publishing journal, trial size), anaesthetic management (e.g., type of PNB, surgery performed under PNB only or combined with other anaesthetic techniques) and outcomes as stated above.

An extraction form was made using Covidence's data extraction tool, shown in Appendix B. Two authors piloted the data extraction form on five randomly selected full‐text articles to ensure that the data collected effectively fulfilled the review's objectives. The clinical suitability of a testing method for a given PNB lies outside the scope of this review and was therefore not assessed.

### 3.6 Synthesis of Results

Data are presented in narrative form or using tables. Proportions are presented with corresponding 95% confidence intervals (CIs). Continuous variables are presented as means with corresponding standard deviations (SDs) for data with normal distributions, and with medians and interquartile ranges (IQRs) for non‐normally distributed data. Subgroup analyses were performed on the following two outcomes:

- The proportion of RCTs that state that PNBs were tested.
- The proportion of RCTs describing how the PNBs were tested.

Subgroup analyses, pre‐defined in the study protocol, were performed based on the following criteria:

- Publishing journal in the upper 50th percentile of Impact Factor, according to the Web of Science 2024 Journal Impact Factor (yes vs. no) [20].
- Published within the last 5 years (after 2019) (yes vs. no).
- PNB as the sole anaesthetic for surgery (yes vs. no).
- ‘Large’ trials (over 100 participants) (yes vs. no).
- Trials with a prospective, publicly accessible protocol (yes vs. no).
- Trials in which the PNB was the primary intervention or comparator (yes vs. no).

Chi‐square tests were used to compare subgroups.

## 4 Results

**FIGURE 1:**
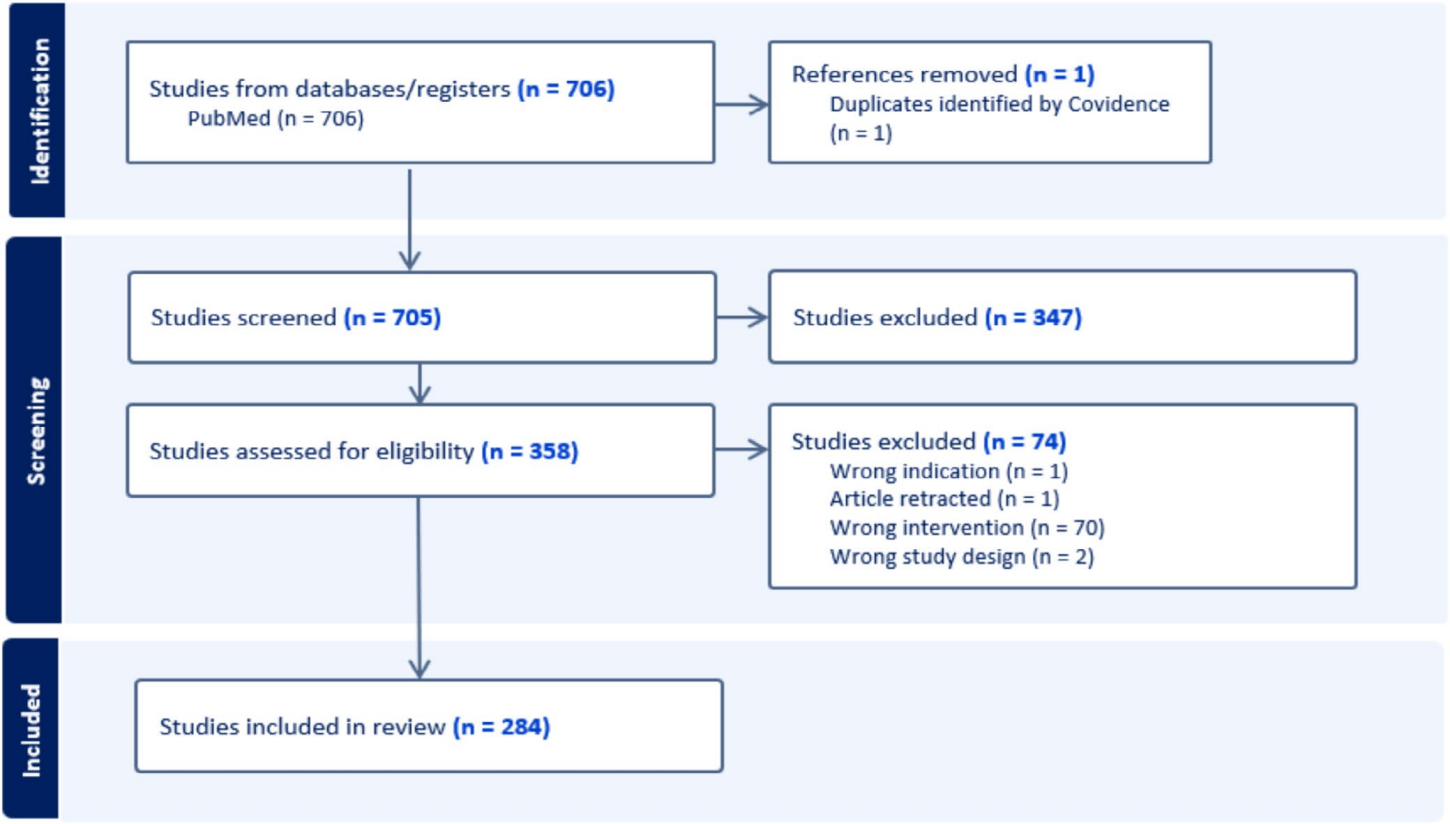
Preferred reporting items for systematic reviews and meta‐analyses flow diagram. Latest search performed on February 20, 2025.

Our search identified 706 studies. Following removal of duplicates and abstract screening, 358 studies were assessed in full‐text screening, of which 284 were included (Figure 1).

### 4.1 Study Characteristics

Trial characteristics are summarised in Table 2. The majority of trials included fewer than 100 patients (227/284, 80%), were published between 2014 and 2019 (175/284, 62%) and approximately half had a prospectively registered, publicly accessible protocol (156/284, 55%). Of the 284 included trials, 162 (57%) investigated upper limb blocks and 121 (43%) investigated lower limb blocks, with one trial including both [22]. Most included trials had a PNB as the primary intervention or comparator (256/284, 90%). Included trials are individually listed in Table S1.

**TABLE 2:** Trial characteristics.

Trials	*N* = 284, *n* (%)
Year of publication	
2014–2019	175 (62)
2020–2025	109 (38)
Publishing journal	
Anesthesiology	18 (6)
British Journal of Anaesthesia	14 (5)
Anaesthesia	17 (6)
Regional Anaesthesia and Pain Medicine	64 (23)
Journal of Clinical Anaesthesia	16 (6)
Anaesthesia & Analgesia	18 (6)
European Journal of Anaesthesiology	22 (8)
Korean Journal of Anesthesiology	15 (5)
Anaesthesia Critical Care & Pain Medicine	4 (1)
Canadian Journal of Anaesthesia	11 (4)
Minerva Anestesiologica	15 (5)
Journal of Anaesthesia	20 (7)
BMC Anesthesiology	39 (14)
Journal of Clinical Monitoring and Computing	2 (1)
Acta Anaesthesiologica Scandinavica	7 (2)
Anaesthesia and Intensive Care	2 (1)
Number of patients randomised	
1–50	73 (26)
51–100	154 (54)
> 100	57 (20)
Trial location (continent and most frequent countries)	
Africa	11 (4)
Egypt	11 (4)
Asia	102 (36)
China	34 (12)
Japan	9 (3)
South Korea	28 (10)
Turkey	9 (3)
Other	22 (8)
Europe	85 (30)
Belgium	10 (4)
France	15 (5)
Italy	10 (4)
Switzerland	14 (5)
Other	36 (13)
North America	81 (28)
Canada	36 (13)
United States of America	45 (16)
Oceania	3 (1)
Australia	3 (1)
South America	10 (4)
Brazil	1 (0)
Chile	9 (3)
Block placement and most common block types	
Upper limb	163 (57)
Interscalene brachial plexus block	74 (26)
Supraclavicular brachial plexus block	43 (15)
Infraclavicular brachial plexus block	26 (9)
Axillary brachial plexus block	27 (9)
Lower limb	122 (43)
Femoral nerve block	54 (19)
Saphenous nerve block	47 (17)
Sciatic nerve block	32 (11)
Popliteal sciatic nerve block	31 (11)

*Note:* Category totals may exceed the number of included trials due to some trials representing data for more than one characteristic.

### 4.2 Review Outcomes

Amongst the included trials, 215/284 (76%, 95% CI 71%–81%) stated that all PNBs performed in the trial were tested, with 210/284 trials (74%, 95% CI 69%–79%) describing the test methods (Table 3). Of the 215 trials stating that all PNBs were tested, 204 (95%, 95% CI 91%–97%) reported using sensory testing and 157 (73%, 95% CI 67%–79%) reported using motor testing, with 151 (70%, 95% CI 64%–76%) reporting both sensory and motor testing. Amongst the trials that reported testing, graded scales (e.g., assessing block quality using a scale with three or more ordered categories) or quantitative measurements such as maximum voluntary isometric contraction were used to assess sensory blocks in 128/204 trials (63%, 95% CI 56%–69%) and motor blocks in 122/157 trials (78%, 95% CI 71%–84%).

**TABLE 3:** Review outcomes.

	*N* = 284, *n* (%, 95% CI)		
	Yes	No	Partial^a^
PNB tested	215 (76, 71–81)	55 (19, 15–24)	14 (5, 2–7)
PNB test described	210 (74, 69–79)	60 (21, 16–26)	14 (5, 2–7)
PNB success criteria defined	164 (58, 52–63)	112 (39, 34–45)	8 (3, 1–5)
PNB success rate reported	164 (58, 52–63)	112 (39, 34–45)	8 (3, 1–5)
Management of data from unsuccessful PNBs described	128 (45, 39–51)	149 (52, 47–58)	7 (2, 1–5)

	*N* = 215, *n* (%, 95% CI)		
	Yes	No	Partial^a^
Sensory function used in testing	204 (95, 91–97)	10 (47, 25–84)	1 (0, 0–3)
Motor function used in testing	157 (73, 67–79)	54 (25, 20–31)	4 (2, 1–5)

Abbreviations: 95% CI, 95% confidence interval; PNB, peripheral nerve block.

^a^ Trials that included more than one PNB but only reported relevant outcome data for some (not all) of the PNBs were classified as partial.

Success criteria (e.g., a certain threshold on a grading scale) for PNBs were specified in 164/284 (58%, 95% CI 52%–63%) of trials, and the same number of trials reported their success rates. Of note, four trials reported a success rate without defining criteria for a successful block [23, 24, 25, 26]. In studies reporting success rates, the median success rate of PNBs was 98% (range 15–100%, IQR 92%–100%). Under half (128/284, 45%, 95% CI 39%–51%) of included trials reported how data from unsuccessful blocks were managed in the statistical analyses.

### 4.3 Subgroup Analyses

Subgroup analyses are shown in Tables 4a and 4b. Trials in which the PNB was the primary intervention or comparator had statistically significantly higher rates of reporting PNB testing and describing how PNBs were tested than trials where the PNB was not the primary intervention or comparator. There were no significant differences in other subgroups, both regarding reporting PNB testing and describing how PNBs were tested.

**TABLE 4a:** Proportion of RCTs that state that PNBs were tested, subgroup analyses.

Subgroup	Sub‐group	*N*	PNB tested (*n* (%, 95% CI))			*p* Yes vs (No and partial^a^)
			Yes	No	Partial^a^	
Publishing journal in the upper 50th percentile of impact factor	Yes	184	137 (74, 68–80)	37 (20, 14–26)	10 (5, 2–9)	*p* = 0.60
	No	100	78 (78, 70–86)	18 (18, 10–26)	4 (4, 0–8)	
Published after 2019	Yes	109	79 (72, 64–81)	25 (23, 15–31)	5 (5, 1–9)	*p* = 0.39
	No	175	136 (78, 72–84)	30 (17, 12–23)	9 (5, 2–8)	
PNB as the sole anaesthetic for surgery	Yes	76	64 (84, 76–92)	8 (11, 4–17)	4 (5, 0–10)	*p* = 0.06
	No	208	151 (73, 67–79)	47 (23, 17–28)	10 (5, 2–8)	
‘Large’ trials (> 100 participants)	Yes	57	47 (82, 73–92)	8 (14, 5–23)	2 (4, 0–8)	*p* = 0.25
	No	227	168 (74, 68–80)	47 (21, 15–26)	12 (5, 2–8)	
Prospective, publicly accessible protocol	Yes	156	116 (74, 68–81)	33 (21, 15–28)	8 (5, 2–9)	*p* = 0.59
	No	128	99 (77, 70–85)	23 (18, 11–25)	6 (5, 1–8)	
Trials in which the PNB was the primary intervention or comparator	Yes	256	199 (78, 73–83)	44 (17, 13–22)	13 (5, 2–8)	*p* = 0.03
	No	28	16 (57, 39–75)	11 (39, 21–57)	1 (4, 0–10)	

Abbreviations: 95% CI, 95% confidence interval; PNB, peripheral nerve block; RCT, randomised controlled trial.

^a^ Trials that included more than one PNB but only reported relevant outcome data for some (not all) of the PNBs were classified as partial.

**TABLE 4b:** Proportion of RCTs that describe how PNBs were tested, subgroup analyses.

Subgroup	Sub‐group	*N*	PNB test described (*n* (%, 95% CI))			*p* Yes vs (No and partial^a^)
			Yes	No	Partial^a^	
Publishing journal in the upper 50th percentile of impact factor	Yes	184	135 (73, 67–80)	39 (21, 15–27)	10 (5, 2–9)	*p* = 0.87
	No	100	75 (75, 67–83)	21 (21, 13–29)	4 (4, 0–8)	
Published after 2019	Yes	109	78 (72, 63–80)	26 (24, 16–32)	5 (5, 1–9)	*p* = 0.56
	No	175	132 (75, 69–82)	34 (19, 14–25)	9 (5, 2–8)	
PNB as the sole anaesthetic for surgery	Yes	76	63 (83, 74–91)	9 (12, 5–19)	4 (5, 0–10)	*p* = 0.05
	No	208	147 (71, 64–76)	51 (25, 19–30)	10 (5, 2–8)	
‘Large’ trials (> 100 participants)	Yes	57	47 (82, 73–92)	8 (14, 5–23)	2 (4, 0–8)	*p* = 0.14
	No	227	163 (72, 66–78)	52 (23, 17–28)	12 (5, 2–8)	
Prospective, publicly accessible protocol	Yes	156	116 (74, 68–81)	32 (21, 14–27)	8 (5, 2–9)	*p* = 0.97
	No	128	94 (73, 66–81)	28 (22, 15–29)	6 (5, 1–8)	
Trials in which the PNB was the primary intervention or comparator	Yes	256	197 (77, 72–82)	46 (18, 13–23)	13 (5, 2–8)	*p* = 0.001
	No	28	13 (46, 28–65)	14 (50, 31–69)	1 (4, 0–10)	

Abbreviations: 95% CI, 95% confidence interval; PNB, peripheral nerve block; RCT, randomised controlled trial.

^a^ Trials that included more than one PNB but only reported relevant outcome data for some (not all) of the PNBs were classified as partial.

## 5 Discussion

### 5.1 Reporting of Block Testing and Block Success

We identified 284 RCTs involving PNBs published in major anaesthesia journals between January 2014 and February 2025. Although 76% of trials reported testing of the performed PNBs, a substantial proportion did not. The observed variability in reporting reflects a previously recognised heterogeneity that exists in research on regional anaesthesia [7, 9, 27].

The number of trials reporting definitions of a successful block was lower than those reporting testing, with approximately two in five trials not reporting the criteria for a successful nerve block. Reporting whether blocks were tested and how successful blocks were defined are essential for assessment of results as well as trial comparability and reproducibility. Furthermore, clear descriptions of testing and success criteria are crucial to clinical implementation of results. Without transparency on these key components, interpretation of outcomes such as postoperative pain and opioid consumption may be futile. Proposed classification systems for failed PNBs may help to standardise reporting [28, 29].

In a 2022 Delphi study conducted to develop a core outcome set for regional anaesthesia and facilitate intertrial comparison, cold sensory testing 30 min after a block was selected as a core outcome [7]. Of the 42 trials published following the publication of this outcome set, 31% (13/42) did not report any form of sensory testing. Data on timing and modality of sensory testing were not recorded in this review, so the number of trials not adhering to this core outcome is likely higher.

### 5.2 Interpretation of Success Rates

The median success rate across included trials was 98%. By comparison, a 2009 study using data from more than 8000 PNBs administered in a clinical context reported a lower success rate of 89% [30]. A potential explanation for this discrepancy is that PNB administration in a research context does not necessarily reflect real‐life clinical practise. For example, a trial protocol may dictate that only experienced clinicians will administer PNBs for included patients, whereas in clinical contexts, PNBs may be administered by clinicians with a broader range of experience. The widespread adoption of ultrasound guidance in contemporary performance of PNBs may also lead to higher success rates [8]. Some degree of reporting bias may also explain the high success rate we found; in our review, a minority of trials reported how data from unsuccessful blocks were managed in statistical analyses. If patients with an unsuccessful block were excluded from trial analyses, this would clearly skew the reported success rate and trial results. Trials should therefore report how data from unsuccessful blocks are managed in the clinical trial setup, and how data from these cases were analysed, for example, using either intention‐to‐treat or per‐protocol analyses.

One included study reported a low success rate of 15%; the authors attributed this to low local anaesthetic volume and a short 15‐min interval between block placement and testing [31]. Whilst these specific details were not part of our data extraction, they illustrate how factors such as technique and timing can affect the reported success rates of PNBs, and hence the generalisability and clinical implications of the results. Drawing conclusions from RCTs comparing PNBs to other anaesthetic interventions may therefore not be possible if the success rate of the PNBs is unknown or skewed due to suboptimal timing or dosing. For example, postoperative pain is a frequently cited outcome in RCTs involving PNBs [32]. Interpretation of this outcome assumes that the administered PNBs provided analgesia as expected. This further highlights the importance of trials reporting how they test blocks, how they define a successful nerve block, the block's success rate and how unsuccessful blocks were handled in statistical analyses.

### 5.3 Trials Without PNBs as the Primary Intervention or Comparator

We included trials where participants received a PNB without this being the trial's primary intervention or comparator. For example, two trials assessed gabapentin and prolonged‐release oxycodone‐naloxone, respectively, for postoperative pain in patients undergoing total knee arthroplasty with femoral and sciatic nerve blocks in addition to spinal anaesthesia [33, 34]. In both trials, block success rate would directly influence outcomes, as a successful PNB would likely reduce postoperative pain, regardless of group allocation. Despite the PNB not being the primary focus of these trials, reporting of block testing, criteria for block success, and success rates remain essential for valid interpretation of trial results. However, this subgroup of trials had significantly lower reporting rates of PNB testing compared to the trials in which PNBs were the primary intervention or comparator.

### 5.4 Strengths and Limitations

This review has several strengths. It was conducted according to a pre‐defined, published protocol and adhered to established methodological frameworks and guidelines. Data screening and extraction for each trial were performed independently by two reviewers, reducing the risk of selection and extraction bias. By limiting the search to RCTs published in the last decade, the review provides an up‐to‐date overview of current research practises in the testing and reporting of peripheral nerve blocks. The large number of included studies further strengthens the robustness of the findings.

This review also has limitations. The search was limited to a single database (PubMed) and included only studies published in a select number of high impact factor peer‐reviewed journals of anaesthesia. These restrictions may have introduced selection bias and limited the generalisability of findings. However, the decision to focus the search on selected, specialty‐relevant journals was made to ensure that included trials would be of high methodological quality, thus preventing results from being skewed by trials published in less established journals, which may not have the same methodological standards. Furthermore, it was thought that potential issues with reporting of PNB testing in high impact factor journals highlighted by this review would be just as, if not more, prevalent in lower impact factor journals. All included journals were indexed on PubMed, which is why the search was not carried out on other databases. Although we recognise the selection bias introduced by the limited selection of journals, we believe that the risk of omission of high‐quality RCTs that would have changed the conclusion is minor. The exclusion of non‐English language trials may have resulted in the omission of relevant trials conducted in other languages. As is typical in scoping reviews, we did not assess the methodological quality of included studies, which means variations in study rigour were not accounted for in the analysis. Data collected regarding testing methodology was limited to whether testing was carried out using sensory or motor modalities and whether graded scales or quantifiable measures were used. This decision was made to provide consistency in the collected data and ensure the review's outcomes could be meaningfully answered, as collecting detailed information on factors such as specific test methodology, timing and conditions would have introduced substantial heterogeneity. However, this also limits the conclusions that can be drawn regarding the variability that exists in testing methodology. Additionally, whilst data extraction was conducted by two independent reviewers for each trial, consensus was reached by a single author, which may have introduced unintentional bias.

### 5.5 Future Implications

Future trials in regional anaesthesia should include clear descriptions of how PNBs are tested and how successful blocks are defined. Consistent use of systematic structures, such as core outcome sets or classification systems for failed PNBs, may help standardise reporting in RCTs, facilitating intertrial comparison and the clinical application of trial findings.

## 6 Conclusions

This scoping review investigated contemporary practise in testing methodology reported in RCTs involving PNBs from major anaesthetic journals. Although most trials do report testing PNBs, about one in four did not. Furthermore, approximately two in five trials did not define what constituted a successful block or report rates of success. Lack of consistent and transparent test methodology poses challenges when comparing trials and performing meta‐analyses, and in translating trial findings into clinical practise. Consistent use of systematic reporting is advised.

## Author Contributions

All authors meet the recommendations for authorship defined by The International Committee of Medical Journal Editors. A.‐X.R.B., K.W.L., L.H.L., and A.K.N. conceived the study concept and design. A.‐X.R.B., R.L.K., M.T.S., S.Y., R.H.F.B., J.E.G., and S.W. conducted title, abstract, and full‐text screening and data extraction. A.‐X.R.B. carried out data analysis and wrote the first draft of this manuscript with support from A.‐S.L.J. L.H.L. and A.K.N. provided project supervision. All authors read and approved the final manuscript.

## Funding

The authors have nothing to report.

## Ethics Statement

Ethics approval was not required for this scoping review.

## Conflicts of Interest

The authors declare no conflicts of interest.

## Supporting information


**Table S1:** Details of included studies.

## Data Availability

All data supporting the findings of this study are available within the article and its Supporting Information. Raw data are available from the corresponding author upon reasonable request.

## Appendix A See Table A1

**TABLE A1:** Search string.

Domain 1: Nerves and plexuses	Domain 2: Nerve blocks/local anaesthesia	Domain 3: Journals	Domain 4: RCTs
(‘brachial plexus’ [MeSH Terms] OR	((((‘local anesth*’ [Title/Abstract] OR	((((((((((((((((((((‘Anaesthesia’ [Journal]) OR	(‘Randomised Controlled Trials as Topic’ [Mesh]) OR
‘cervical plexus’ [MeSH Terms] OR	‘local anaesth*’ [Title/Abstract] OR	(‘British journal of anaesthesia’ [Journal])) OR	(‘comparative study’ [Publication Type] OR
‘lumbosacral plexus’ [MeSH Terms] OR	‘anaesthesia, local’ [MeSH Terms] OR	(‘Anesthesiology’ [Journal])) OR	‘randomised controlled trial’ [Publication Type] OR
‘thoracic nerves’ [MeSH Terms] OR	‘nerve block’ [MeSH Terms] OR	(‘Journal of clinical anaesthesia’ [Journal])) OR	‘random*’ [Title/Abstract] OR
‘femoral nerve’ [MeSH Terms] OR	‘nerve block*’ [Title/Abstract]) OR	(‘Anaesthesia and analgesia’ [Journal])) OR	‘comparative’ [Title/Abstract])
‘intercostal nerves’ [MeSH Terms] OR	(‘nerve block’ [Title/Abstract:~3])) OR	(‘Anaesthesia Critical Care & Pain Medicine’ [Journal])) OR	
‘median nerve’ [MeSH Terms] OR	(‘nerve blockade’ [Title/Abstract:~3])) OR	(‘Regional anaesthesia and pain medicine’ [Journal])) OR	
‘obturator nerve’ [MeSH Terms] OR	(‘plexus block’ [Title/Abstract:~3])) OR	(‘Best Practise & Research‐Clinical Anaesthesiology’ [Journal])) OR	
‘peroneal nerve’ [MeSH Terms] OR	(‘plexus blockade’ [Title/Abstract:~3])	(‘Canadian journal of anaesthesia Journal canadien d'anesthésie’ [Journal])) OR	
‘tibial nerve’ [MeSH Terms] OR		(‘European journal of anaesthesiology’ [Journal])) OR	
‘radial nerve’ [MeSH Terms] OR		(‘Minerva anestesiologica’ [Journal])) OR	
‘sciatic nerve’ [MeSH Terms] OR		(‘Korean journal of anesthesiology’ [Journal])) OR	
‘superior cervical ganglion’ [MeSH Terms] OR		(‘Journal of anaesthesia’ [Journal])) OR	
‘sural nerve’ [MeSH Terms] OR		(‘Current opinion in anaesthesiology’ [Journal])) OR	
‘ulnar nerve’ [MeSH Terms] OR		(‘BMC anesthesiology’ [Journal])) OR	
‘brachial plexus’ [Title/Abstract:~3] OR		(‘Journal of clinical monitoring and computing’ [Journal])) OR	
‘cervical plexus’ [Title/Abstract:~3] OR		(‘Acta anaesthesiologica Scandinavica’ [Journal])) OR	
‘lumbosacral plexus’ [Title/Abstract:~3] OR		(‘Anaesthesia and intensive care’ [Journal]))	
‘femoral nerve’ [Title/Abstract:~3] OR			
‘femoral nerves’ [Title/Abstract:~3] OR			
‘intercostal nerve’ [Title/Abstract:~3] OR			
‘intercostal nerves’ [Title/Abstract:~3] OR			
‘median nerve’ [Title/Abstract:~3] OR			
‘median nerves’ [Title/Abstract:~3] OR			
‘obturator nerve’ [Title/Abstract:~3] OR			
‘obturator nerves’ [Title/Abstract:~3] OR			
‘peroneus nerve’ [Title/Abstract:~3] OR			
‘peroneus nerves’ [Title/Abstract:~3] OR			
‘tibial nerve’ [Title/Abstract:~3] OR			
‘tibial nerves’ [Title/Abstract:~3] OR			
‘radial nerve’ [Title/Abstract:~3] OR			
‘radial nerves’ [Title/Abstract:~3] OR			
‘saphenous nerve’ [Title/Abstract:~3] OR			
‘saphenous nerves’ [Title/Abstract:~3] OR			
‘sciatic nerve’ [Title/Abstract:~3] OR			
‘sciatic nerves’ [Title/Abstract:~3] OR			
‘sural nerve’ [Title/Abstract:~3] OR			
‘sural nerves’ [Title/Abstract:~3] OR			
‘ulnar nerve’ [Title/Abstract:~3] OR			
‘ulnar nerves’ [Title/Abstract:~3] OR			
‘cervical ganglion’ [Title/Abstract:~3]) OR			
(((‘Sciatic Nerve’ [MeSH Terms] OR			
‘Femoral Nerve’ [MeSH Terms]) AND			
‘Nerve Block’ [MeSH Terms]) OR			
(‘psoas compartment block*’ [Title/Abstract] OR			
‘adductor canal block*’ [Title/Abstract] OR			
‘saphenous nerve block*’ [Title/Abstract] OR			
‘saphenous block*’ [Title/Abstract] OR			
‘femoral nerve block*’ [Title/Abstract] OR			
‘femoral block*’ [Title/Abstract] OR			
‘sciatic nerve block*’ [Title/Abstract] OR			
‘sciatic block*’ [Title/Abstract]) OR			
(‘axillary brachial plexus block*’ [Title/Abstract] OR			
‘axillary plexus block*’ [Title/Abstract] OR			
‘axillary nerve block*’ [Title/Abstract] OR			
‘infraclavicular brachial plexus block*’ [Title/Abstract] OR			
‘infraclavicular plexus block*’ [Title/Abstract] OR			
‘infraclavicular nerve block*’ [Title/Abstract] OR			
‘supraclavicular brachial plexus block*’ [Title/Abstract] OR			
‘supraclavicular plexus block*’ [Title/Abstract] OR			
‘supraclavicular nerve block*’ [Title/Abstract] OR			
‘interscalene brachial plexus block*’ [Title/Abstract] OR			
‘interscalene plexus block*’ [Title/Abstract] OR			
‘interscalene nerve block*’ [Title/Abstract]))			
