# Supplementary material for: Testing of Pre‐Operative Peripheral Nerve Blocks in Randomised Controlled Trials: A Scoping Review
Source: Acta Anaesthesiol Scand. 2026 Feb 26;70(4):e70211. doi: 10.1111/aas.70211 (PMC12946583; doi:10.1111/aas.70211)
Supplement: Supplementary file 1 — Table S1: Details of included studies. [file AAS-70-0-s001.docx]

**Supplementary Table 1: Details of Included Studies**

| First author | Title | Year | Journal | Country | Trial registry number |
| --- | --- | --- | --- | --- | --- |
| Abbal | Enhanced visual acuity with echogenic needles in ultrasound-guided axillary brachial plexus block: a randomized, comparative, observer-blinded study | 2015 | Minerva Anestesiologica | France | EudraCT 2010-A00297-32 |
| Abdallah | The analgesic effects of proximal, distal, or no sciatic nerve block on posterior knee pain after total knee arthroplasty: a double-blind placebo-controlled randomized trial | 2014 | Anesthesiology | Canada | N/A |
| Abdallah | Intravenous dexamethasone and perineural dexamethasone similarly prolong the duration of analgesia after supraclavicular brachial plexus block: a randomized, triple-arm, double-blind, placebo-controlled trial | 2015 | Regional Anesthesia and Pain Medicine | Canada | N/A |
| Abdallah | IV and Perineural Dexmedetomidine Similarly Prolong the Duration of Analgesia after Interscalene Brachial Plexus Block: A Randomized, Three-arm, Triple-masked, Placebo-controlled Trial | 2016 | Anesthesiology | Canada | NCT02225054 |
| Abdallah | Adductor Canal Block Provides Noninferior Analgesia and Superior Quadriceps Strength Compared with Femoral Nerve Block in Anterior Cruciate Ligament Reconstruction | 2016 | Anesthesiology | Canada | NCT01791036 |
| Abdallah | Opioid- and Motor-sparing with Proximal, Mid-, and Distal Locations for Adductor Canal Block in Anterior Cruciate Ligament Reconstruction: A Randomized Clinical Trial | 2019 | Anesthesiology | Canada | NCT02554864 |
| Abdallah | Subomohyoid Anterior Suprascapular Block versus Interscalene Block for Arthroscopic Shoulder Surgery: A Multicenter Randomized Trial | 2020 | Anesthesiology | Canada | NCT02517437 |
| Abdelghany | Superficial cervical plexus block alone or combined with interscalene brachial plexus block in surgery for clavicle fractures: a randomized clinical trial | 2021 | Minerva Anestesiologica | Egypt | PACTR201702002032348 |
| Abdelhamid | Nalbuphine as an adjuvant to 0.25% levobupivacaine in ultrasound-guided supraclavicular block provided prolonged sensory block and similar motor block durations (RCT) | 2018 | Journal of Anesthesia | Egypt | PACTR201705002264264 |
| Abdulatif | The effects of perineural dexmedetomidine on the pharmacodynamic profile of femoral nerve block: a dose-finding randomised, controlled, double-blind study | 2016 | Anaesthesia | Egypt | NCT02089932 |
| Aboelfadl | Infiltration of local anesthetic in the Interspace between the popliteal artery and capsule of the posterior knee "IPACK block" versus adductor canal block "ACB" for pain relief after open wedge high tibial osteotomy: a randomized clinical trial | 2024 | Minerva Anestesiologica | Egypt | NCT05271188 |
| Abo-Zeid Salim | Intravenous versus perineural dexmedetomidine as adjuvant in adductor canal block for total knee arthroplasty | 2023 | Korean Journal of Anesthesiology | Egypt | NCT04266145 |
| Admiraal | The effectiveness of ambulatory continuous popliteal sciatic nerve blockade on patient-reported overall benefit of analgesia in patients undergoing foot or ankle surgery (CAREFREE trial); a randomized, open label, non-inferiority trial | 2024 | Journal of Clinical Anesthesia | Netherlands | NL9382 |
| Aguilera | 0.25% bupivacaine-1% lidocaine vs 0.5% bupivacaine for ultrasound-guided infraclavicular brachial plexus block: a randomized controlled trial | 2024 | Regional Anesthesia and Pain Medicine | Chile | NCT05834023 |
| Aksoy | Continuous spinal anaesthesia versus ultrasound-guided combined psoas compartment-sciatic nerve block for hip replacement surgery in elderly high-risk patients: a prospective randomised study | 2014 | BMC Anesthesiology | Turkey | ACTRN12614000658617 |
| Albrecht | Reduced hemidiaphragmatic paresis with extrafascial compared with conventional intrafascial tip placement for continuous interscalene brachial plexus block: a randomized, controlled, double-blind trial | 2017 | British Journal of Anaesthesia | Switzerland | NCT02433561 |
| Albrecht | Dose-response relationship of perineural dexamethasone for interscalene brachial plexus block: a randomised, controlled, triple-blind trial | 2019 | Anaesthesia | Belgium | EudraCT 2017-002815-32 |
| Albrecht | A randomised controlled trial of intravenous dexmedetomidine added to dexamethasone for arthroscopic rotator cuff repair and duration of interscalene block | 2023 | Anaesthesia | France | NCT04394481 |
| Aliste | A randomized comparison between intravenous and perineural dexamethasone for ultrasound-guided axillary block | 2017 | Canadian Journal of Anesthesia | Canada, Thailand | NCT02629835 |
| Aliste | A Randomized Comparison Between Interscalene and Small-Volume Supraclavicular Blocks for Arthroscopic Shoulder Surgery | 2018 | Regional Anesthesia and Pain Medicine | Chile | NCT03224884 |
| Aliste | Randomized comparison between perineural dexamethasone and dexmedetomidine for ultrasound-guided infraclavicular block | 2019 | Regional Anesthesia and Pain Medicine | Chile | NCT03610893 |
| Aliste | Randomized comparison between interscalene and costoclavicular blocks for arthroscopic shoulder surgery | 2019 | Regional Anesthesia and Pain Medicine | Chile | NCT03411343 |
| Aliste | Randomized comparison between perineural dexamethasone and combined perineural dexamethasone-dexmedetomidine for ultrasound-guided infraclavicular block | 2022 | Regional Anesthesia and Pain Medicine | Chile | NCT04875039 |
| Ambrosoli | A randomised controlled trial comparing two popliteal nerve catheter tip positions for postoperative analgesia after day-case hallux valgus repair | 2016 | Anaesthesia | Italy | NCT02029404 |
| Ambrosoli | A randomised controlled trial of intrathecal blockade versus peripheral nerve blockade for day-case knee arthroscopy | 2016 | Anaesthesia | Italy | N/A |
| Amundson | A Three-arm Randomized Clinical Trial Comparing Continuous Femoral Plus Single-injection Sciatic Peripheral Nerve Blocks versus Periarticular Injection with Ropivacaine or Liposomal Bupivacaine for Patients Undergoing Total Knee Arthroplasty | 2017 | Anesthesiology | USA | NCT02223364 |
| Aoyama | Continuous quadratus lumborum block and femoral nerve block for total hip arthroplasty: a randomized study | 2020 | Journal of Anesthesia | Japan | UMIN000028928 |
| Aoyama | Incidence and effects of postoperative migration of interscalene catheter tips placed using ultrasound-guided anterior and posterior approaches | 2021 | Journal of Anesthesia | Japan | UMIN000007541 |
| Arab | Ultrasound-guided supraclavicular brachial plexus block: single versus triple injection technique for upper limb arteriovenous access surgery | 2014 | Anesthesia & Analgesia | Saudi Arabia | N/A |
| Arjun | Ultrasound-guided interscalene block combined with intermediate or superficial cervical plexus block for clavicle surgery: A randomised double blind study | 2020 | European Journal of Anaesthesiology | India | CTRI/2018/05/013785 |
| Arnuntasupakul | A randomized trial comparing axillary block versus targeted intracluster injection supraclavicular block for upper limb surgery | 2015 | Canadian Journal of Anesthesia | Canada | N/A |
| Auyong | A Double-Blind Randomized Comparison of Continuous Interscalene, Supraclavicular, and Suprascapular Blocks for Total Shoulder Arthroplasty | 2017 | Regional Anesthesia and Pain Medicine | USA | NCT02117778 |
| Auyong | Comparison of Anterior Suprascapular, Supraclavicular, and Interscalene Nerve Block Approaches for Major Outpatient Arthroscopic Shoulder Surgery: A Randomized, Double-blind, Noninferiority Trial | 2018 | Anesthesiology | USA | NCT02287142 |
| Bao | Effect of local anesthetic volume (20 mL vs 30 mL ropivacaine) on electromyography of the diaphragm and pulmonary function after ultrasound-guided supraclavicular brachial plexus block: a randomized controlled trial | 2019 | Regional Anesthesia and Pain Medicine | China | ChiCTR-IND-17012166 |
| Bao | Dexmedetomidine prolongs the duration of local anesthetics when used as an adjuvant through both perineural and systemic mechanisms: a prospective randomized double-blinded trial | 2022 | BMC Anesthesiology | China | ChiCTR-IOR-17011149 |
| Barrington | A Randomized Controlled Trial of Ultrasound Versus Nerve Stimulator Guidance for Axillary Brachial Plexus Block | 2016 | Regional Anesthesia and Pain Medicine | Australia | ACTRN12605000750684 |
| Bergmann | Phrenic nerve block caused by interscalene brachial plexus block: breathing effects of different sites of injection | 2016 | BMC Anesthesiology | Germany | DRKS00009908 |
| Beylacq | Perifascial plane versus perineural approaches for ultrasound-guided axillary block: go to the simplest? | 2020 | Minerva Anestesiologica | France | N/A |
| Bharti | The Analgesic Efficacy of Dexmedetomidine as an Adjunct to Local Anesthetics in Supraclavicular Brachial Plexus Block: A Randomized Controlled Trial | 2015 | Anesthesia & Analgesia | India | CTRI/2013/12/004209 |
| Bharti | Comparison of ultrasound-guided supraclavicular, infraclavicular and below-C6 interscalene brachial plexus block for upper limb surgery: a randomised, observer-blinded study | 2015 | Anaesthesia and Intensive Care | India | CTRI/2014/08/004822 |
| Bielka | Psoas compartment block efficacy and safety for perioperative analgesia in the elderly with proximal femur fractures: a randomized controlled study | 2021 | BMC Anesthesiology | Ukraine | NCT04648332 |
| Bingül | Comparing the clinical features of lateral and medial approaches of costoclavicular technique versus traditional lateral sagittal technique as infraclavicular brachial plexus block methods: a randomized controlled trial | 2024 | BMC Anesthesiology | Turkey | NCT05260736 |
| Biswas | Relative Contributions of Adductor Canal Block and Intrathecal Morphine to Analgesia and Functional Recovery After Total Knee Arthroplasty: A Randomized Controlled Trial | 2018 | Regional Anesthesia and Pain Medicine | Canada | NCT02411149 |
| Bjørn | Effect of Perineural Dexamethasone on the Duration of Single Injection Saphenous Nerve Block for Analgesia After Major Ankle Surgery: A Randomized, Controlled Study | 2017 | Regional Anesthesia and Pain Medicine | Denmark | NCT02346110 |
| Bjørn | The Importance of the Saphenous Nerve Block for Analgesia Following Major Ankle Surgery: A Randomized, Controlled, Double-Blind Study | 2018 | Regional Anesthesia and Pain Medicine | Denmark | NCT02697955 |
| Black | The analgesic efficacy of the transversalis fascia plane block in iliac crest bone graft harvesting: a randomized controlled trial | 2019 | Korean Journal of Anesthesiology | Canada | NCT01133730 |
| Blanco | Retroclavicular vs Infraclavicular block for brachial plexus anesthesia: a multi-centric randomized trial | 2019 | BMC Anesthesiology | Canada | NCT02913625 |
| Bombardieri | A comparison of two different concentrations and infusion rates of ropivacaine in perineural infusion administered at the same total dose for analgesia after foot and ankle surgery: a randomized, double blinded, controlled study | 2019 | Minerva Anestesiologica | USA | N/A |
| Bravo | A multicenter, randomized comparison between 2, 5, and 8 mg of perineural dexamethasone for ultrasound-guided infraclavicular block | 2019 | Regional Anesthesia and Pain Medicine | Canada, Chile, Thailand | TCTR20150624001 |
| Brenner | Efficacy of axillary versus infraclavicular brachial plexus block in preventing tourniquet pain: A randomised trial | 2019 | European Journal of Anaesthesiology | Ireland | NCT02714738 |
| Brookes | Comparative evaluation of the visibility and block characteristics of a stimulating needle and catheter vs an echogenic needle and catheter for sciatic nerve block with a low-frequency ultrasound probe | 2015 | British Journal of Anaesthesia | Canada | NCT01492660 |
| Cappelleri | Effect of local anesthetic dilution on the onset time and duration of double-injection sciatic nerve block: a prospective, randomized, blinded evaluation | 2014 | Anesthesia & Analgesia | Italy | EudraCT 2013-004633-32 |
| Cappelleri | A randomised controlled comparison between stimulating and standard catheters for lumbar plexus block | 2015 | Anaesthesia | Italy | N/A |
| Cappelleri | Effects of the Intraneural and Subparaneural Ultrasound-Guided Popliteal Sciatic Nerve Block: A Prospective, Randomized, Double-Blind Clinical and Electrophysiological Comparison | 2016 | Regional Anesthesia and Pain Medicine | Italy | NCT01987128 |
| Chalifoux | Low dose intravenous dexamethasone (4 mg and 10 mg) significantly prolongs the analgesic duration of single-shot interscalene block after arthroscopic shoulder surgery: a prospective randomized placebo-controlled study | 2017 | Canadian Journal of Anesthesia | Canada | NCT02412657 |
| Chan | Addition of Liposomal Bupivacaine to Standard Bupivacaine versus Standard Bupivacaine Alone in the Supraclavicular Brachial Plexus Block: A Randomized Controlled Trial | 2024 | Anesthesiology | China | NCT05118399 |
| Charvin | Effects of adding a combined femoral and sciatic nerve block with levobupivacaine and clonidine to general anaesthesia in femoropopliteal bypass surgery: A randomised, double-blind, controlled trial | 2020 | European Journal of Anaesthesiology | France | NCT01785693 |
| Chassery | Total knee arthroplasty under quadruple nerve block with ropivacaine 0.32%: effect of addition of intravenous dexmedetomidine to intravenous dexamethasone on analgesic duration | 2021 | Regional Anesthesia and Pain Medicine | France | NCT03834129 |
| Chen | Lipid Emulsion Pretreatment Decreased the Maximum Total and Free Plasma Concentration of Levobupivacaine for Femoral and Sciatic Nerve Block in Below-Knee Fracture Surgery | 2018 | Regional Anesthesia and Pain Medicine | China | ChiCTR-TRC-14005203 |
| Chen | Application of ultrasound-guided single femoral triangle and adductor canal block in arthroscopic knee surgery: a prospective, double-blind, randomized clinical study | 2024 | BMC Anesthesiology | China | ChiCTR2300068765 |
| Cho | Double-injection perivascular ultrasound-guided axillary brachial plexus block according to needle positioning: 12 versus 6 o'clock position of the axillary artery | 2014 | Korean Journal of Anesthesiology | South Korea | N/A |
| Choi | Optimizing Pain and Rehabilitation After Knee Arthroplasty: A Two-Center, Randomized Trial | 2016 | Anesthesia & Analgesia | Canada | NCT01616836 |
| Choquet | Subparaneural versus circumferential extraneural injection at the bifurcation level in ultrasound-guided popliteal sciatic nerve blocks: a prospective, randomized, double-blind study | 2014 | Regional Anesthesia and Pain Medicine | France | EudraCT 2010-A00289-30 |
| Choromanski | The effect of continuous interscalene brachial plexus block with 0.125% bupivacaine vs 0.2% ropivacaine on pain relief, diaphragmatic motility, and ventilatory function | 2015 | Journal of Clinical Anesthesia | USA | NCT02059070 |
| Clarke | Perioperative gabapentin reduces 24 h opioid consumption and improves in-hospital rehabilitation but not post-discharge outcomes after total knee arthroplasty with peripheral nerve block | 2014 | British Journal of Anaesthesia | Canada | N/A |
| Clement | Clinical effectiveness of single dose of intravenous dexamethasone on the duration of ropivacaine axillary brachial plexus block: the randomized placebo-controlled ADEXA trial | 2019 | Regional Anesthesia and Pain Medicine | France | NCT02862327 |
| Coudray | Combination of real-time needle-tip pressure sensing and minimal intensity stimulation limits unintentional intraneural injection during an ultrasound-guided peripheral nerve block procedure: A randomized, parallel group, controlled trial | 2021 | Journal of Clinical Anesthesia | France | NCT02737137 |
| Desmet | A randomised controlled trial of intravenous dexamethasone combined with interscalene brachial plexus blockade for shoulder surgery | 2015 | Anaesthesia | Belgium | N/A |
| Dhir | A randomised comparison between ultrasound and nerve stimulation for infraclavicular catheter placement | 2016 | Anaesthesia | Canada | N/A |
| Dhir | A Comparison of Combined Suprascapular and Axillary Nerve Blocks to Interscalene Nerve Block for Analgesia in Arthroscopic Shoulder Surgery: An Equivalence Study | 2016 | Regional Anesthesia and Pain Medicine | Canada | NCT01730573 |
| Dhir | Infraclavicular and supraclavicular approaches to brachial plexus for ambulatory elbow surgery: A randomized controlled observer-blinded trial | 2018 | Journal of Clinical Anesthesia | Canada | NCT02677506 |
| Dieguez-Garcia | Comparison of the effectiveness of circumferential versus non-circumferential spread in median and ulnar nerve blocks. A double-blind randomized clinical trial | 2020 | Regional Anesthesia and Pain Medicine | Spain | EudraCT 2011-002608-34, NCT01603680 |
| Elkassabany | The Risk of Falls After Total Knee Arthroplasty with the Use of a Femoral Nerve Block Versus an Adductor Canal Block: A Double-Blinded Randomized Controlled Study | 2016 | Anesthesia & Analgesia | USA | NCT02314832 |
| Elmer | Comparing bupivacaine alone to liposomal bupivacaine plus bupivacaine in interscalene blocks for total shoulder arthroplasty: a randomized, non-inferiority trial | 2023 | Regional Anesthesia and Pain Medicine | USA | NCT04974385 |
| Fei | Continuous block at the proximal end of the adductor canal provides better analgesia compared to that at the middle of the canal after total knee arthroplasty: a randomized, double-blind, controlled trial | 2020 | BMC Anesthesiology | China | NCT03942133 |
| Fenten | Effect of local anesthetic concentration, dose and volume on the duration of single-injection ultrasound-guided axillary brachial plexus block with mepivacaine: a randomized controlled trial | 2015 | BMC Anesthesiology | Netherlands | NTR3648 |
| Ferré | Hemidiaphragmatic paralysis following ultrasound-guided anterior vs. posterior suprascapular nerve block: a double-blind, randomised control trial | 2020 | Anaesthesia | France | NCT03352687 |
| Finneran | Suture-method versus Through-the-needle Catheters for Continuous Popliteal-sciatic Nerve Blocks: A Randomized Clinical Trial | 2020 | Anesthesiology | USA | NCT03442036 |
| Fisker | Combined saphenous and sciatic catheters for analgesia after major ankle surgery: a double-blinded randomized controlled trial | 2015 | Canadian Journal of Anesthesia | Denmark | NCT01445210 |
| Flaherty | Comparing liposomal bupivacaine plus bupivacaine to bupivacaine alone in interscalene blocks for rotator cuff repair surgery: a randomized clinical trial | 2022 | Regional Anesthesia and Pain Medicine | USA | NCT03587584 |
| Flohr-Madsen | A randomised placebo-controlled trial examining the effect on hand supination after the addition of a suprascapular nerve block to infraclavicular brachial plexus blockade | 2016 | Anaesthesia | Norway | NCT02035774 |
| Fritsch | Dexmedetomidine added to ropivacaine extends the duration of interscalene brachial plexus blocks for elective shoulder surgery when compared with ropivacaine alone: a single-center, prospective, triple-blind, randomized controlled trial | 2014 | Regional Anesthesia and Pain Medicine | Austria | NCT01557270 |
| Fujino | Migration rate of proximal adductor canal block catheters placed parallel versus perpendicular to the nerve after total knee arthroplasty: a randomized controlled study | 2023 | Regional Anesthesia and Pain Medicine | Japan | UMIN000045374 |
| Gadsden | The relative analgesic value of a femoral nerve block versus adductor canal block following total knee arthroplasty: a randomized, controlled, double-blinded study | 2020 | Korean Journal of Anesthesiology | USA | NCT03395990 |
| Gargano | A randomized clinical trial comparing different combination of peripheral nerve blocks for intraoperative analgesia in patients on antithrombotic drugs undergoing hip fracture surgery: pericapsular nerve group (PENG) block versus femoral and obturator nerve block | 2024 | Minerva Anestesiologica | Italy | NCT06457880 |
| Georgiadis | Ultrasound-guided supraclavicular vs. retroclavicular block of the brachial plexus: comparison of ipsilateral diaphragmatic function: A randomised clinical trial | 2021 | European Journal of Anaesthesiology | USA | NCT02631122 |
| Gerber | Clinical effect of normal saline injectate into interscalene nerve block catheters given within one hour of local anesthetic bolus on analgesia and hemidiaphragmatic paralysis | 2021 | Regional Anesthesia and Pain Medicine | USA | NCT03677778 |
| Ghazaly | Comparison of the efficacy of two doses of dexmedetomidine as an adjunct to levobupivacaine in infraclavicular brachial plexus block: prospective double-blinded randomized controlled trial | 2022 | BMC Anesthesiology | Egypt | NCT04729868 |
| Gi | Effects of local infiltration analgesia for posterior knee pain after total knee arthroplasty: comparison with sciatic nerve block | 2014 | Journal of Anesthesia | Japan | N/A |
| Gianesello | The influence of interscalene block technique on adverse hemodynamic events | 2014 | Journal of Anesthesia | Italy | N/A |
| Gianesello | Respiratory effect of interscalene brachial plexus block vs combined infraclavicular plexus block with suprascapular nerve block for arthroscopic shoulder surgery | 2018 | Journal of Clinical Anesthesia | Italy | N/A |
| Gleicher | Comparison of migration rates between traditional and tunneled adductor canal block catheters: a randomized controlled trial | 2024 | Regional Anesthesia and Pain Medicine | Canada | NCT05313269 |
| Grape | Retroclavicular vs supraclavicular brachial plexus block for distal upper limb surgery: a randomised, controlled, single-blinded trial | 2019 | British Journal of Anaesthesia | Switzerland | NCT02641613 |
| Grape | Characteristics of a single versus multiple-injection axillary brachial plexus block: A single-blinded randomised, clinical trial | 2021 | European Journal of Anaesthesiology | Switzerland | NCT03378323 |
| Grape | Impact of an extrafascial versus intrafascial injection for supraclavicular brachial plexus block on respiratory function: a randomized, controlled, double-blind trial | 2022 | Regional Anesthesia and Pain Medicine | Switzerland | NCT03957772 |
| Grass | Timing of intravenous dexamethasone and analgesia after brachial plexus block: a randomized, double-blind, placebo-controlled trial | 2024 | Regional Anesthesia and Pain Medicine | Canada | NCT03394820 |
| Guilley | Femoral nerve inguinal approach versus proximal femoral triangle ap proach for continuous regional analgesia in active rehabilitation after total knee arthroplasty: A prospective, randomised study | 2022 | Anaesthesia Critical Care & Pain Medicine | France | NCT02873637 |
| Gunjiyal | Effect of combined versus sequential injection of 2% lidocaine and 0.5% bupivacaine on the onset and duration of supraclavicular brachial plexus block: A double blinded randomised controlled trial | 2021 | Journal of Clinical Anesthesia | India | CTRI/2018/10/016118 |
| Hadzic | Liposome Bupivacaine Femoral Nerve Block for Postsurgical Analgesia after Total Knee Arthroplasty | 2016 | Anesthesiology | USA | NCT01683071 |
| Hanson | Continuous ultrasound-guided adductor canal block for total knee arthroplasty: a randomized, double-blind trial | 2014 | Anesthesia & Analgesia | USA | N/A |
| Harbell | Combined preoperative femoral and sciatic nerve blockade improves analgesia after anterior cruciate ligament reconstruction: a randomized controlled clinical trial | 2016 | Journal of Clinical Anesthesia | USA | NCT01447277 |
| Hattammaru | Reduction of leakage from insertion site during continuous femoral nerve block with catheter-through-needle versus catheter-over-needle technique for postoperative analgesia after total knee arthroplasty: a randomized controlled trial | 2022 | BMC Anesthesiology | Japan | UMIN000021537 |
| Hauritz | The effect of perineural dexamethasone on duration of sciatic nerve blockade: a randomized, double-blind study | 2018 | Acta Anaesthesiologica Scandinavica | Denmark | EudraCT 2014-005383-14 |
| Hay | Comparison of pericapsular nerve group and lateral quadratus lumborum blocks on cumulative opioid consumption after primary total hip arthroplasty: a randomized controlled trial | 2024 | Regional Anesthesia and Pain Medicine | USA | NCT05710107 |
| He | Two different placement paths in popliteal fossa with a novel nerve block needle for postoperative analgesia after foot and ankle surgery | 2018 | Minerva Anestesiologica | China | N/A |
| He | The effect of warming ropivacaine on ultrasound-guided subgluteal sciatic nerve block: a randomized controlled trial | 2023 | BMC Anesthesiology | China | ChiCTR2200064350 |
| Head | Ultrasound-guided saphenous nerve block--within versus distal to the adductor canal: a proof-of-principle randomized trial | 2015 | Canadian Journal of Anesthesia | Canada | N/A |
| Holland | Effect of dexamethasone dose and route on the duration of interscalene brachial plexus block for outpatient arthroscopic shoulder surgery: a randomized controlled trial | 2018 | Canadian Journal of Anesthesia | Canada | NCT02426736 |
| Holmberg | Analgesic effect of intravenous dexamethasone after volar plate surgery for distal radius fracture with brachial plexus block anaesthesia: a prospective, double-blind randomised clinical trial | 2020 | Anaesthesia | Norway | NCT03011905 |
| Hong | Sedation with dexmedetomidine prolongs the analgesic duration of brachial plexus block: a randomised controlled trial | 2019 | Anaesthesia Critical Care & Pain Medicine | South Korea | KCT0002138 |
| Janssen | Blood pressure response to combined general anaesthesia/interscalene brachial plexus block for outpatient shoulder arthroscopy | 2014 | BMC Anesthesiology | Germany | DRKS00005295 |
| Jeong | A comparison of motor stimulation threshold in ultrasound-guided interscalene brachial plexus block for arthroscopic shoulder surgery: a randomized trial | 2016 | Canadian Journal of Anesthesia | South Korea | KCT0000582 |
| Jo | Comparison of the ulnar nerve blockade between intertruncal and corner pocket approaches for supraclavicular block: a randomized controlled trial | 2021 | Korean Journal of Anesthesiology | South Korea | KCT0005268 |
| Johnston | Effect of combining peri-hamstring injection or anterior obturator nerve block on the analgesic efficacy of adductor canal block for anterior cruciate ligament reconstruction: a randomised controlled trial | 2020 | British Journal of Anaesthesia | Canada | NCT01868282 |
| Kalimeris | Dislocation rates of perineural catheters placed either perpendicular or parallel to the femoral nerve: A randomised controlled trial | 2020 | European Journal of Anaesthesiology | Switzerland | NCT03693755 |
| Kampitak | Motor-sparing effect of iPACK (interspace between the popliteal artery and capsule of the posterior knee) block versus tibial nerve block after total knee arthroplasty: a randomized controlled trial | 2020 | Regional Anesthesia and Pain Medicine | Thailand | TCTR20180206002 |
| Kampitak | The analgesic efficacy of anterior femoral cutaneous nerve block in combination with femoral triangle block in total knee arthroplasty: a randomized controlled trial | 2021 | Korean Journal of Anesthesiology | Thailand | TCTR20191209004 |
| Kanadli | Comparison of the efficacy of femoral nerve block and fascia iliaca compartment block in patients with total knee replacement | 2018 | Minerva Anestesiologica | Turkey | N/A |
| Kang | Effective Dose of Intravenous Dexmedetomidine to Prolong the Analgesic Duration of Interscalene Brachial Plexus Block: A Single-Center, Prospective, Double-Blind, Randomized Controlled Trial | 2018 | Regional Anesthesia and Pain Medicine | South Korea | KCT0002119 |
| Kang | Reduced Hemidiaphragmatic Paresis With a "Corner Pocket" Technique for Supraclavicular Brachial Plexus Block: Single-Center, Observer-Blinded, Randomized Controlled Trial | 2018 | Regional Anesthesia and Pain Medicine | South Korea | KCT0001769 |
| Kang | Superior Trunk Block Provides Noninferior Analgesia Compared with Interscalene Brachial Plexus Block in Arthroscopic Shoulder Surgery | 2019 | Anesthesiology | South Korea | KCT0002802 |
| Kang | Improvement in postoperative pain control by combined use of intravenous dexamethasone with intravenous dexmedetomidine after interscalene brachial plexus block for arthroscopic shoulder surgery: A randomised controlled trial | 2019 | European Journal of Anaesthesiology | South Korea | KCT0002569 |
| Kastelik | Local infiltration anaesthesia versus sciatic nerve and adductor canal block for fast-track knee arthroplasty: A randomised controlled clinical trial | 2019 | European Journal of Anaesthesiology | Germany | NCT03114306 |
| Kim | A randomized comparison of long-axis and short-axis imaging for in-plane ultrasound-guided popliteal-sciatic perineural catheter insertion | 2014 | Journal of Anesthesia | USA | NCT01459523 |
| Kim | Adductor canal block versus femoral nerve block for total knee arthroplasty: a prospective, randomized, controlled trial | 2014 | Anesthesiology | USA | NCT01333943 |
| Kim | A comparison of ultrasound alone vs ultrasound with nerve stimulation guidance for continuous femoral nerve block in patients undergoing total knee arthroplasty | 2016 | Journal of Clinical Anesthesia | South Korea | KCT0001196 |
| Kim | Combined use of dexmedetomidine and propofol in monitored anesthesia care: a randomized controlled study | 2017 | BMC Anesthesiology | South Korea | KCT0001284 |
| Kim | A comparison of ultrasound-guided interscalene and supraclavicular blocks for post-operative analgesia after shoulder surgery | 2017 | Acta Anaesthesiologica Scandinavica | South Korea | NCT02014766 |
| Kim | Superior Trunk Block: A Phrenic-sparing Alternative to the Interscalene Block: A Randomized Controlled Trial | 2019 | Anesthesiology | USA | NCT03272139 |
| Kim | Effect of intravenous dexamethasone on the duration of postoperative analgesia for popliteal sciatic nerve block: a randomized, double-blind, placebo-controlled study | 2021 | Korean Journal of Anesthesiology | South Korea | KCT0002486 |
| Kim | Interscalene Brachial Plexus Block with Liposomal Bupivacaine versus Standard Bupivacaine with Perineural Dexamethasone: A Noninferiority Trial | 2022 | Anesthesiology | USA | NCT04047446 |
| Kim | Effect of remifentanil on post-operative analgesic consumption in patients undergoing shoulder arthroplasty after interscalene brachial plexus block: a randomized controlled trial | 2022 | Journal of Anesthesia | South Korea | NCT04236323 |
| Kim | Comparison between low-volume local anesthetic with intravenous dexamethasone and conventional volume without dexamethasone for superior trunk block after arthroscopic shoulder surgery: a randomized controlled non-inferiority trial | 2023 | Regional Anesthesia and Pain Medicine | South Korea | KCT0005998 |
| Kim | A comparison of the continuous supraclavicular brachial plexus block using the proximal longitudinal oblique approach and the interscalene brachial plexus block for arthroscopic shoulder surgery: A randomised, controlled, double-blind trial | 2023 | European Journal of Anaesthesiology | South Korea | KCT0004759 |
| Kim | Comparison of the Analgesic Efficacy between Arthroscopically Placed Continuous Suprascapular Nerve Block and Ultrasound-guided Continuous Superior Trunk Block: A Double-blinded Randomized Controlled Trial | 2023 | Anesthesiology | South Korea | KCT0005500 |
| Kjelstrup | MRI of axillary brachial plexus blocks: a randomised controlled study | 2014 | European Journal of Anaesthesiology | Norway | NCT01033006 |
| Koh | Use of hyaluronidase as an adjuvant to ropivacaine to reduce axillary brachial plexus block onset time: a prospective, randomised controlled study | 2015 | Anaesthesia | South Korea | KCT0000964 |
| Koh | A randomised controlled trial comparing continuous supraclavicular and interscalene brachial plexus blockade for open rotator cuff surgery | 2016 | Anaesthesia | South Korea | N/A |
| Kuchálik | Does local infiltration analgesia reduce peri-operative inflammation following total hip arthroplasty? A randomized, double-blind study | 2017 | BMC Anesthesiology | Sweden | EudraCT 2012-003875-20 |
| Kull | Femoral vs sciatic nerve block to provide analgesia after medial open wedge high tibial osteotomy in the setting of multimodal analgesia: A randomized, controlled, single-blinded trial | 2024 | Journal of Clinical Anesthesia | Switzerland | NCT05728294 |
| Kwon | The effect of dexmedetomidine as an adjuvant to ropivacaine on the bispectral index for supraclavicular brachial plexus block | 2015 | Korean Journal of Anesthesiology | South Korea | N/A |
| Layera | Single- versus double-injection costoclavicular block: a randomized comparison | 2020 | Regional Anesthesia and Pain Medicine | Chile | NCT03595514 |
| Lee | Comparison between two different concentrations of a fixed dose of ropivacaine in interscalene brachial plexus block for pain management after arthroscopic shoulder surgery: a randomized clinical trial | 2021 | Korean Journal of Anesthesiology | South Korea | KCT0003785 |
| Lee | Does intravenous patient-controlled analgesia or continuous block prevent rebound pain following infraclavicular brachial plexus block after distal radius fracture fixation? A prospective randomized controlled trial | 2023 | Korean Journal of Anesthesiology | South Korea | KCT0003404 |
| Lee | Costoclavicular block as a diaphragm-sparing nerve block for shoulder surgery: a randomized controlled trial | 2025 | Korean Journal of Anesthesiology | South Korea | KCT0002376 |
| Leurcharusmee | A Multicenter Randomized Comparison Between Intravenous and Perineural Dexamethasone for Ultrasound-Guided Infraclavicular Block | 2016 | Regional Anesthesia and Pain Medicine | Canada, Thailand | TCTR20150624001 |
| Leurcharusmee | A randomized comparison between costoclavicular and paracoracoid ultrasound-guided infraclavicular block for upper limb surgery | 2017 | Canadian Journal of Anesthesia | Canada, Thailand | TCTR20160525001 |
| Li | Regional haemodynamic changes after selective block of the four principal nerves in the arm: A double-blind randomised controlled study | 2016 | European Journal of Anaesthesiology | China | NCT02139982 |
| Li | Dose-response studies of Ropivacaine in blood flow of upper extremity after supraclavicular block: a double-blind randomized controlled study | 2017 | BMC Anesthesiology | China | NCT02139982 |
| Li | Adding dexmedetomidine to ropivacaine for femoral nerve block inhibits local inflammatory response | 2017 | Minerva Anestesiologica | China | N/A |
| Liang | Comparison between pericapsular nerve group (PENG) block with lateral femoral cutaneous nerve block and supra-inguinal fascia iliaca compartment block (S-FICB) for total hip arthroplasty: a randomized controlled trial | 2023 | Journal of Anesthesia | China | ChiCTR2100051521 |
| Lim | Preoperative interscalene brachial plexus block aids in perioperative temperature management during arthroscopic shoulder surgery | 2016 | Korean Journal of Anesthesiology | South Korea | N/A |
| Lim | Randomized, controlled trial comparing respiratory and analgesic effects of interscalene, anterior suprascapular, and posterior suprascapular nerve blocks for arthroscopic shoulder surgery | 2020 | Korean Journal of Anesthesiology | Singapore | NCT03277326 |
| Lin | Pericapsular nerve group (PENG) block provides improved short-term analgesia compared with the femoral nerve block in hip fracture surgery: a single-center double-blinded randomized comparative trial | 2021 | Regional Anesthesia and Pain Medicine | Australia | NL8043 |
| Liu | Is there a dose response of dexamethasone as adjuvant for supraclavicular brachial plexus nerve block? A prospective randomized double-blinded clinical study | 2015 | Journal of Clinical Anesthesia | USA | NCT01690663 |
| Liu | Analgesic effect of Ropivacaine combined with Dexmedetomidine on brachial plexus block | 2018 | BMC Anesthesiology | China | ChiCTR1800017372 |
| Liu | Low-dose dexmedetomidine as a perineural adjuvant for postoperative analgesia: a randomized controlled trial | 2022 | BMC Anesthesiology | China | NCT02630290 |
| López | Effective volumes of 1.5% mepivacaine with different sodium concentration for ultrasound guided popliteal block | 2017 | Journal of Clinical Anesthesia | Spain | ISRCTN13419503 |
| Lu | Comparison of lumbar plexus block using the short axis in-plane method at the plane of the transverse process and at the articular process: a randomized controlled trial | 2018 | BMC Anesthesiology | China | ChiCTR-INR-15007505 |
| Luo | Double-injection technique assisted by a nerve stimulator for ultrasound-guided supraclavicular brachial plexus block results in better distal sensory-motor block: A randomised controlled trial | 2017 | European Journal of Anaesthesiology | China | ChiCTR-IOR-15007588 |
| Luo | Effects of the costoclavicular block versus interscalene block in patients undergoing arthroscopic shoulder surgery under monitored anesthesia care: a randomized, prospective, non-inferiority study | 2023 | Korean Journal of Anesthesiology | China | ChiCTR2000040841 |
| Machi | Discharge Readiness after Tricompartment Knee Arthroplasty: Adductor Canal versus Femoral Continuous Nerve Blocks-A Dual-center, Randomized Trial | 2015 | Anesthesiology | USA | NCT01759277 |
| Magazzeni | Ultrasound-Guided Selective Versus Conventional Block of the Medial Brachial Cutaneous and the Intercostobrachial Nerves: A Randomized Clinical Trial | 2018 | Regional Anesthesia and Pain Medicine | France | NCT02940847 |
| Manassero | Oral prolonged-release oxycodone/naloxone offers equivalent analgesia to intravenous morphine patient-controlled analgesia after total knee replacement. A randomized controlled trial | 2018 | Minerva Anestesiologica | Italy | N/A |
| Mangar | Knee strength retention and analgesia with continuous perineural fentanyl infusion after total knee replacement: randomized controlled trial | 2014 | Journal of Anesthesia | USA | NCT01620047 |
| Marashi | Naloxone added to bupivacaine or bupivacaine-fentanyl prolongs motor and sensory block during supraclavicular brachial plexus blockade: a randomized clinical trial | 2015 | Acta Anaesthesiologica Scandinavica | Iran | IRCT201209175140N6 |
| Marian | A Comparison of 2 Ultrasound-Guided Approaches to the Saphenous Nerve Block: Adductor Canal Versus Distal Transsartorial: A Prospective, Randomized, Blinded, Noninferiority Trial | 2015 | Regional Anesthesia and Pain Medicine | USA | NCT02383615 |
| Marty | Obturator nerve block does not provide analgesic benefits in total hip arthroplasty under multimodal analgesic regimen: a randomized controlled trial | 2021 | Regional Anesthesia and Pain Medicine | France | NCT04085640 |
| Marty | Combined proximal or distal nerve blocks for postoperative analgesia after total knee arthroplasty: a randomised controlled trial | 2022 | British Journal of Anaesthesia | France | NCT04499716 |
| McHardy | Comparison of the effects of perineural or intravenous dexamethasone on low volume interscalene brachial plexus block: a randomised equivalence trial | 2020 | British Journal of Anaesthesia | Canada | NCT02322242 |
| Meco | Can we gain an advantage by combining distal median, radial and ulnar nerve blocks with supraclavicular block? A randomized controlled study | 2015 | Journal of Anesthesia | Turkey | NCT01989312 |
| Monahan | Continuous Popliteal Sciatic Blocks: Does Varying Perineural Catheter Location Relative to the Sciatic Bifurcation Influence Block Effects? A Dual-Center, Randomized, Subject-Masked, Controlled Clinical Trial | 2016 | Anesthesia & Analgesia | USA | NCT01229696 |
| Montgomery | Effect of Nerve Stimulation Use on the Success Rate of Ultrasound-Guided Subsartorial Saphenous Nerve Block: A Randomized Controlled Trial | 2017 | Regional Anesthesia and Pain Medicine | Canada | NCT02382744 |
| Moustafa | Randomized comparative study between two different techniques of intercostobrachial nerve block together with brachial plexus block during superficialization of arteriovenous fistula | 2018 | Journal of Anesthesia | Egypt | PACTR201708002462294 |
| Mouzi | Plasma concentrations of ropivacaine following ultrasound-guided or nerve-stimulator-guided femoral nerve block: A prospective randomised study | 2016 | Anaesthesia Critical Care & Pain Medicine | USA | N/A |
| Muñoz-Leyva | No Benefits of Adding Dexmedetomidine, Ketamine, Dexamethasone, and Nerve Blocks to an Established Multimodal Analgesic Regimen after Total Knee Arthroplasty | 2022 | Anesthesiology | Canada | NCT03954379 |
| Maalouf | Prospective, Randomized Double-Blind Study: Does Decreasing Interscalene Nerve Block Volume for Surgical Anesthesia in Ambulatory Shoulder Surgery Offer Same-Day Patient Recovery Advantages? | 2016 | Regional Anesthesia and Pain Medicine | USA | NCT01701115 |
| Nader | Single-Dose Adductor Canal Block With Local Infiltrative Analgesia Compared With Local Infiltrate Analgesia After Total Knee Arthroplasty: A Randomized, Double-Blind, Placebo-Controlled Trial | 2016 | Regional Anesthesia and Pain Medicine | USA | NCT02100579 |
| Neuts | Selective Suprascapular and Axillary Nerve Block Versus Interscalene Plexus Block for Pain Control After Arthroscopic Shoulder Surgery: A Noninferiority Randomized Parallel-Controlled Clinical Trial | 2018 | Regional Anesthesia and Pain Medicine | Belgium | NCT02415088 |
| Nickl | Impact of self-coiling catheters for continuous popliteal sciatic block on postoperative pain level and dislocation rate: a randomized controlled trial | 2022 | BMC Anesthesiology | Germany | DRKS00020938 |
| Nijs | Ultrasound-guided axillary brachial plexus block versus distal peripheral forearm nerve block for hand and wrist surgery: a randomised controlled trial | 2023 | British Journal of Anaesthesia | Belgium | NCT04678765 |
| Novello-Siegenthaler | Ultrasound-guided continuous femoral nerve block: a randomized trial on the influence of femoral nerve catheter orifice configuration (six-hole versus end-hole) on post-operative analgesia after total knee arthroplasty | 2018 | BMC Anesthesiology | Switzerland | NCT03376178 |
| Ochroch | Analgesic efficacy of adding the IPACK block to a multimodal analgesia protocol for primary total knee arthroplasty | 2020 | Regional Anesthesia and Pain Medicine | USA | NCT03703206 |
| Olive | A randomised controlled trial comparing three analgesia regimens following total knee joint replacement: continuous femoral nerve block, intrathecal morphine or both | 2015 | Anaesthesia and Intensive Care | Australia | ACTRN12606000421538 |
| Oliver-Fornies | A randomised controlled trial in patients undergoing arthroscopic shoulder surgery comparing interscalene block with either 10 ml or 20 ml levobupivacaine 0.25 | 2022 | Anaesthesia | Spain | NCT04385966 |
| Olofsson | Duration of analgesia after forefoot surgery compared between an ankle and a sciatic nerve block at the popliteal crease: A randomised controlled single-blinded trial | 2024 | European Journal of Anaesthesiology | Switzerland | NCT03683342 |
| Ozcan | Comparison of the efficacy of different concentrations and volumes of levobupivacaine in axillary brachial plexus blockade | 2014 | Minerva Anestesiologica | Turkey | N/A |
| Ozturk | Bispectral index-guided general anaesthesia in combination with interscalene block reduces desflurane consumption in arthroscopic shoulder surgery: a clinical comparison of bupivacaine versus levobupivacaine | 2015 | BMC Anesthesiology | Turkey | ACTRN12613000381785 |
| Ozturk | Comparison of the coracoid and retroclavicular approaches for ultrasound-guided infraclavicular brachial plexus block | 2017 | Journal of Anesthesia | Turkey | NCT02673086 |
| Palhais | Extrafascial injection for interscalene brachial plexus block reduces respiratory complications compared with a conventional intrafascial injection: a randomized, controlled, double-blind trial | 2016 | British Journal of Anaesthesia | Switzerland | NCT02074397 |
| Paulou | Analgesic efficacy of selective tibial nerve block versus partial local infiltration analgesia for posterior pain after total knee arthroplasty: a randomized, controlled, triple-blinded trial | 2023 | Anaesthesia Critical Care & Pain Medicine | Switzerland | NCT03698006 |
| Perov | Effective low dosage of mepivacaine in ultrasound-guided axillary nerve block: a double-blinded, randomized clinical trial of efficacy in patients undergoing distal upper extremity surgery | 2014 | Journal of Clinical Anesthesia | USA | NCT01485653 |
| Petrar | Hemidiaphragmatic paralysis following ultrasound-guided supraclavicular versus infraclavicular brachial plexus blockade: a randomized clinical trial | 2015 | Regional Anesthesia and Pain Medicine | Canada | N/A |
| Petroff | Differential lung ventilation assessed by electrical impedance tomography in ultrasound-guided anterior suprascapular nerve block vs. interscalene brachial plexus block: A patient and assessor-blind, randomised controlled trial | 2020 | European Journal of Anaesthesiology | Germany | DRKS00011787 |
| Pulitanò | Ropivacaine and magnesium sulfate in sciatic nerve block at the popliteal level: randomized double-blind study | 2024 | Minerva Anestesiologica | Italy | EudraCT 2013-004863-30 |
| Quek | Adding a PECS II block for proximal arm arteriovenous access - a randomised study | 2018 | Acta Anaesthesiologica Scandinavica | Singapore | NCT02331030 |
| R | Equal mixture of 2% lidocaine with adrenaline and 0.5% bupivacaine 20 mL provided faster onset of complete conduction blockade during ultrasound-guided supraclavicular brachial plexus block than 20 mL of 0.5% bupivacaine alone: a randomized double-blinded clinical trial | 2024 | Regional Anesthesia and Pain Medicine | India | CTRI/2020/11/029359 |
| Rahangdale | The effects of perineural versus intravenous dexamethasone on sciatic nerve blockade outcomes: a randomized, double-blind, placebo-controlled study | 2014 | Anesthesia & Analgesia | USA | NCT01616173 |
| Ranganath | Effect of two volumes (10 and 30 ml) of lidocaine 2% and epinephrine on the duration of axillary brachial plexus block: A randomised controlled trial | 2022 | European Journal of Anaesthesiology | Ireland | NCT03163472 |
| Renard | Respiratory impact of local anaesthetic volume after an interscalene brachial plexus block with an extrafascial injection: a randomised controlled double-blinded trial | 2025 | British Journal of Anaesthesia | Switzerland | NCT04726280 |
| Reynolds | Analgesic Benefit of Pectoral Nerve Block II Blockade for Open Subpectoral Biceps Tenodesis: A Randomized, Prospective, Double-Blinded, Controlled Trial | 2019 | Anesthesia & Analgesia | USA | NCT02741713 |
| Rhyner | A randomised controlled trial of shoulder block vs. interscalene brachial plexus block for ventilatory function after shoulder arthroscopy | 2020 | Anaesthesia | Switzerland | NCT02916342 |
| Rhyner | Single-bolus injection of local anesthetic, with or without continuous infusion, for interscalene brachial plexus block in the setting of multimodal analgesia: a randomized controlled unblinded trial | 2023 | Regional Anesthesia and Pain Medicine | Switzerland | NCT04394130 |
| Rodríguez Prieto | Low-concentration distal nerve blocks with 0.125% levobupivacaine versus systemic analgesia for ambulatory trapeziectomy performed under axillary block: a randomized controlled trial | 2018 | Minerva Anestesiologica | Spain | NCT01597479 |
| Rosenfeld | Perineural versus intravenous dexamethasone as adjuncts to local anaesthetic brachial plexus block for shoulder surgery | 2016 | Anaesthesia | USA | N/A |
| Rothe | A randomized controlled trial evaluating the impact of selective axillary nerve block after arthroscopic subacromial decompression | 2020 | BMC Anesthesiology | Denmark | NCT01463865 |
| Rousseau-Saine | The Effect of Adductor Canal Block on Knee Extensor Muscle Strength 6 Weeks After Total Knee Arthroplasty: A Randomized, Controlled Trial | 2018 | Anesthesia & Analgesia | Canada | NCT02166710 |
| Safa | Comparison of the Analgesic Duration of 0.5% Bupivacaine With 1:200,000 Epinephrine Versus 0.5% Ropivacaine Versus 1% Ropivacaine for Low-Volume Ultrasound-Guided Interscalene Brachial Plexus Block: A Randomized Controlled Trial | 2021 | Anesthesia & Analgesia | Canada | NCT02643563 |
| Sakae | Dexamethasone as a ropivacaine adjuvant for ultrasound-guided interscalene brachial plexus block: A randomized, double-blinded clinical trial | 2017 | Journal of Clinical Anesthesia | Brazil | RBR-86mhm2 |
| Sakai | Equivalence of postoperative quadriceps strength during 1 or 0.5 mg ml(-1) levobupivacaine administration for continuous femoral nerve block following total knee arthroplasty: A double-blinded, randomised controlled trial | 2015 | European Journal of Anaesthesiology | Japan | JPRN-UMIN000006893 |
| Salih Sevdi | Lateral Trendelenburg with the injected side down after the block improves the efficacy of the axillary approach to brachial plexus block | 2014 | Journal of Anesthesia | Turkey | N/A |
| Samerchua | Randomized comparison between ultrasound-guided proximal and distal approaches of intercostobrachial nerve block as an adjunct to supraclavicular brachial plexus block for upper arm arteriovenous access procedures | 2024 | Regional Anesthesia and Pain Medicine | Thailand | TCTR20200730006 |
| Saporito | The effect of continuous popliteal sciatic nerve block on unplanned postoperative visits and readmissions after foot surgery--a randomised, controlled study comparing day-care and inpatient management | 2014 | Anaesthesia | Switzerland | N/A |
| Sato | Continuous versus single-injection sciatic nerve block added to continuous femoral nerve block for analgesia after total knee arthroplasty: a prospective, randomized, double-blind study | 2014 | Regional Anesthesia and Pain Medicine | Japan | UMIN000005789 |
| Schoenmakers | The effects of adding epinephrine to ropivacaine for popliteal nerve block on the duration of postoperative analgesia: a randomized controlled trial | 2015 | BMC Anesthesiology | The Netherlands | NTR3330 |
| Schwartz | A phase 3 active-controlled trial of liposomal bupivacaine via sciatic nerve block in the popliteal fossa after bunionectomy | 2024 | Journal of Clinical Anesthesia | USA | NCT05157841 |
| Sciard | Postoperative analgesia after surgical repair of distal radius fracture: a randomized comparison between distal peripheral nerve blockade and surgical site infiltration | 2023 | Minerva Anestesiologica | France | NCT02693288 |
| Seering | Comparison of the effect of three different adjuvants on the analgesic duration of single injection interscalene brachial plexus block: a prospective, randomized, triple blinded clinical trial | 2019 | Regional Anesthesia and Pain Medicine | United States of America | NCT03117140 |
| Sellbrant | Supraclavicular block with Mepivacaine vs Ropivacaine, their impact on postoperative pain: a prospective randomised study | 2021 | BMC Anesthesiology | Sweden | NCT03749174 |
| Sermeus | Thermal quantitative sensory testing to assess the sensory effects of three local anesthetic solutions in a randomized trial of interscalene blockade for shoulder surgery | 2016 | Canadian Journal of Anesthesia | Belgium | NCT02271867 |
| Sermeus | A low dose of three local anesthetic solutions for interscalene blockade tested by thermal quantitative sensory testing: a randomized controlled trial | 2019 | Journal of Clinical Monitoring and Computing | Belgium | NCT02691442 |
| Sherif | Dexamethasone as adjuvant for femoral nerve block following knee arthroplasty: a randomized, controlled study | 2016 | Acta Anaesthesiologica Scandinavica | Egypt | PACTR201407000850309 |
| Shin | Effective analgesia with ultrasound-guided interscalene brachial plexus block for postoperative pain control after arthroscopic rotator cuff repair | 2014 | Journal of Anesthesia | South Korea | N/A |
| Shokri | Sciatic obturator femoral technique versus spinal anaesthesia in patients undergoing surgery for fixation of open tibial fractures using Ilizarov external fixator. A randomised trial | 2020 | BMC Anesthesiology | Egypt | NCT03450798 |
| Short | Intermittent bolus versus continuous infusion popliteal sciatic nerve block following major foot and ankle surgery: a prospective randomized comparison | 2019 | Regional Anesthesia and Pain Medicine | Canada | NCT02707874 |
| Simonis | The vertical obturator nerve block: A randomised controlled double-blind pilot trial | 2016 | European Journal of Anaesthesiology | Austria | NCT01875289 |
| Sirivanasandha | Adding a low-concentration sciatic nerve block to total knee arthroplasty in patients susceptible to the adverse effects of non-steroidal anti-inflammatory drugs (NSAIDs): a randomized controlled trial | 2021 | BMC Anesthesiology | Thailand | NCT03486548 |
| Sivashanmugam | Randomized Comparison of Extrafascial Versus Subfascial Injection of Local Anesthetic During Ultrasound-Guided Supraclavicular Brachial Plexus Block | 2015 | Regional Anesthesia and Pain Medicine | India | CTRI/2013/12/004180 |
| Sivashanmugam | Ipsilateral hemidiaphragmatic paresis after a supraclavicular and costoclavicular brachial plexus block: A randomised observer blinded study | 2019 | European Journal of Anaesthesiology | India | CTRI/2017/09/009763 |
| Soberón | Ultrasound-guided popliteal sciatic nerve blockade in the severely and morbidly obese: a prospective and randomized study | 2016 | Journal of Anesthesia | USA | NCT01550094 |
| Song | Comparison of dexmedetomidine and epinephrine as an adjuvant to 1% mepivacaine in brachial plexus block | 2014 | Korean Journal of Anesthesiology | South Korea | N/A |
| Song | Comparison of the ultrasound-guided single-injection femoral triangle block versus adductor canal block for analgesia following total knee arthroplasty: a randomized, double-blind trial | 2020 | Journal of Anesthesia | China | ChiCTR-INR-17012716 |
| Songthamwat | Ultrasound-Guided Infraclavicular Brachial Plexus Block: Prospective Randomized Comparison of the Lateral Sagittal and Costoclavicular Approach | 2018 | Regional Anesthesia and Pain Medicine | China | CUHK_CCT00389 |
| Sort | Peripheral nerve block anaesthesia and postoperative pain in acute ankle fracture surgery: the AnAnkle randomised trial | 2021 | British Journal of Anaesthesia | Denmark | EudraCT 2015-001108-76 |
| Stundner | Comparison of tissue distribution, phrenic nerve involvement, and epidural spread in standard- vs low-volume ultrasound-guided interscalene plexus block using contrast magnetic resonance imaging: a randomized, controlled trial | 2016 | British Journal of Anaesthesia | Austria | NCT02175069 |
| Sun | Continuous interscalene versus phrenic nerve-sparing high-thoracic erector spinae plane block for total shoulder arthroplasty: a randomized controlled trial | 2022 | Canadian Journal of Anesthesia | USA | NCT03807505 |
| Sundarathiti | Comparison of continuous femoral nerve block (CFNB/SA) and continuous femoral nerve block with mini-dose spinal morphine (CFNB/SAMO) for postoperative analgesia after total knee arthroplasty (TKA): a randomized controlled study | 2016 | BMC Anesthesiology | Thailand | TCTR20150609003 |
| Swenson | Randomized controlled trial of a simplified adductor canal block performed for analgesia following total knee arthroplasty | 2019 | Regional Anesthesia and Pain Medicine | USA | NCT02786888 |
| Swisser | Plantar Compartment Block Improves Enhanced Recovery after Hallux Valgus Surgery: A Randomized, Comparative, Double-blind Study | 2024 | Anesthesiology | France | NCT03922412 |
| Sztain | Continuous Adductor Canal Versus Continuous Femoral Nerve Blocks: Relative Effects on Discharge Readiness Following Unicompartment Knee Arthroplasty | 2015 | Regional Anesthesia and Pain Medicine | USA | NCT01759277 |
| Sztain | Proximal Versus Distal Continuous Adductor Canal Blocks: Does Varying Perineural Catheter Location Influence Analgesia? A Randomized, Subject-Masked, Controlled Clinical Trial | 2018 | Anesthesia & Analgesia | USA | NCT02523235 |
| Szűcs | A comparison of three techniques (local anesthetic deposited circumferential to vs. above vs. below the nerve) for ultrasound guided femoral nerve block | 2014 | BMC Anesthesiology | Ireland | NCT01527812 |
| Sørensen | Effects of popliteal plexus block after total knee arthroplasty: a randomized clinical trial | 2024 | Regional Anesthesia and Pain Medicine | Denmark | EudraCT 2021-000242-17 |
| Taha | Diaphragm-sparing effect of the infraclavicular subomohyoid block vs low volume interscalene block. A randomized blinded study | 2019 | Acta Anaesthesiologica Scandinavica | UAE | NCT03331237 |
| Tammam | Ultrasound-guided sciatic nerve block: a comparison between four different infragluteal probe and needle alignment approaches | 2014 | Journal of Anesthesia | Egypt | N/A |
| Tammam | Use of a curved needle to facilitate lateral sagittal infraclavicular block performance: a randomized clinical trial | 2019 | Journal of Anesthesia | Egypt | NCT02799576 |
| Techasuk | A randomized comparison between double-injection and targeted intracluster-injection ultrasound-guided supraclavicular brachial plexus block | 2014 | Anesthesia & Analgesia | Canada, Chile | N/A |
| Teunkens | Patient satisfaction with intravenous regional anaesthesia or an axillary block for minor ambulatory hand surgery: A randomised controlled study | 2020 | European Journal of Anaesthesiology | Belgium | EudraCT 2016-002325-11 |
| Tiyaprasertkul | A Randomized Comparison Between Single- and Triple-Injection Subparaneural Popliteal Sciatic Nerve Block | 2015 | Regional Anesthesia and Pain Medicine | Canada, Chile and Thailand | N/A |
| Touil | Evaluation of intraoperative ketamine on the prevention of severe rebound pain upon cessation of peripheral nerve block: a prospective randomised, double-blind, placebo-controlled study | 2022 | British Journal of Anaesthesia | Belgium | NCT04890418 |
| Turan | Novel needle guide reduces time to perform ultrasound-guided femoral nerve catheter placement: A randomised controlled trial | 2017 | European Journal of Anaesthesiology | USA | NCT02080481 |
| Turner | Perineural dexamethasone successfully prolongs adductor canal block when assessed by objective pinprick sensory testing: A prospective, randomized, dose-dependent, placebo-controlled equivalency trial | 2018 | Journal of Clinical Anesthesia | USA | NCT02462148 |
| Tziona | Local infiltration analgesia combined with a standardized multimodal approach including an adductor canal block in total knee arthroplasty: a prospective randomized, placebo-controlled, double-blinded clinical trial | 2018 | Journal of Anesthesia | Greece | NCT03206554 |
| Van Boxstael | Effect of Lidocaine 2% Versus Bupivacaine 0.5% and 1 Versus 2 Dual Separate Injections on Onset and Duration of Ultrasound-Guided Wrist Blocks: A Blinded 2 × 2 Factorial Randomized Clinical Trial | 2022 | Anesthesia & Analgesia | Belgium | EudraCT 2017-003694-34 |
| Vandepitte | Addition of Liposome Bupivacaine to Bupivacaine HCl Versus Bupivacaine HCl Alone for Interscalene Brachial Plexus Block in Patients Having Major Shoulder Surgery | 2017 | Regional Anesthesia and Pain Medicine | Belgium | NCT02554357 |
| Wahal | Femoral artery block (FAB) attenuates thigh tourniquet-induced hypertension: a prospective randomized, double-blind, placebo-controlled trial | 2021 | Regional Anesthesia and Pain Medicine | USA | NCT03390426 |
| Wang | The efficacy of simultaneous bilateral axillary brachial plexus block under the guidance of neurostimulator or ultrasound: a prospective study | 2016 | Journal of Anesthesia | China | ChiCTR-IOC-15006689 |
| Wang | The effect of continuous adductor canal block combined with distal interspace between the popliteal artery and capsule of the posterior knee block for total knee arthroplasty: a randomized, double-blind, controlled trial | 2022 | BMC Anesthesiology | China | ChiCTR2200059139 |
| Wang | Comparison of the efficacy of costoclavicular space brachial plexus blockade with 0.5% versus 0.375% ropivacaine: a randomized, double-blind, single-centre, noninferiority clinical trial | 2023 | Canadian Journal of Anesthesia | China | ChiCTR20000306570 |
| Wang | Comparison of pulmonary function during interscalene block vs. supraclavicular block: a single-center, double-blind, randomized trial | 2023 | BMC Anesthesiology | China | ChiCTR1900028286 |
| Wiegel | Anterior Suprascapular Nerve Block Versus Interscalene Brachial Plexus Block for Shoulder Surgery in the Outpatient Setting: A Randomized Controlled Patient- and Assessor-Blinded Trial | 2017 | Regional Anesthesia and Pain Medicine | Germany | DRKS00009565 |
| Wiesmann | Supplemental single shot femoral nerve block for total hip arthroplasty: impact on early postoperative care, pain management and lung function | 2014 | Minerva Anestesiologica | Germany | DRKS00000752 |
| Wiesmann | Phrenic palsy and analgesic quality of continuous supraclavicular vs. interscalene plexus blocks after shoulder surgery | 2016 | Acta Anaesthesiologica Scandinavica | Germany | DRKS00006147 |
| Wiesmann | Ultrasound-guided single injection versus continuous sciatic nerve blockade on pain management and mobilisation after total knee arthroplasty (CoSinUS trial): A randomised, triple-blinded controlled trial | 2018 | European Journal of Anaesthesiology | Germany | DRKS00010152 |
| Wong | Infraclavicular nerve block reduces postoperative pain after distal radial fracture fixation: a randomized controlled trial | 2020 | BMC Anesthesiology | China | NCT03048214 |
| Woo | Dose-dependency of dexamethasone on the analgesic effect of interscalene block for arthroscopic shoulder surgery using ropivacaine 0.5%: A randomised controlled trial | 2015 | European Journal of Anaesthesiology | South Korea | KCT0001078 |
| Woo | Perineural dexamethasone reduces rebound pain after ropivacaine single injection interscalene block for arthroscopic shoulder surgery: a randomized controlled trial | 2021 | Regional Anesthesia and Pain Medicine | South Korea | KCT0004418 |
| Xu | Ultrasound-guided superficial cervical plexus block combined with clavipectoral fascial plane block or interscalene brachial plexus block in clavicle surgery: a single-centre, double-blind, randomized controlled trial | 2023 | Journal of Clinical Monitoring and Computing | China | ChiCTR2000039383 |
| Xu | Combined femoral artery block and femoral nerve block reduces thigh tourniquet-induced hypertension | 2023 | Journal of Clinical Anesthesia | China | ChiCTR2200060376 |
| YaDeau | Pregabalin and pain after total knee arthroplasty: a double-blind, randomized, placebo-controlled, multidose trial | 2015 | British Journal of Anaesthesia | USA | NCT01333956 |
| YaDeau | Addition of Dexamethasone and Buprenorphine to Bupivacaine Sciatic Nerve Block: A Randomized Controlled Trial | 2015 | Regional Anesthesia and Pain Medicine | USA | NCT02198235 |
| Yamamoto | A prospective, randomized comparison between single- and multiple-injection techniques for ultrasound-guided subgluteal sciatic nerve block | 2014 | Anesthesia & Analgesia | Japan | JPRN-UMIN000005673 |
| Yang | A comparison of posterior and medial cord stimulation for neurostimulation-guided vertical infraclavicular block: a randomized noninferiority clinical trial | 2014 | Anesthesia & Analgesia | South Korea | N/A |
| Yazer | A randomized comparison between infraclavicular block and targeted intracluster injection supraclavicular block | 2015 | Regional Anesthesia and Pain Medicine | Canada | N/A |
| Yektaş | Comparison of sciatic nerve block quality achieved using the anterior and posterior approaches: a randomised trial | 2019 | BMC Anesthesiology | Turkey | N/A |
| Yin | Effect of adductor canal block combined with infiltration between the popliteal artery and posterior capsular of the knee on chronic pain after total knee arthroplasty: a prospective, randomized, double-blind, placebo-controlled trial | 2024 | BMC Anesthesiology | China | ChiCTR2200065300 |
| Yoshida | An Ultrasound-Guided Lateral Approach for Proximal Sciatic Nerve Block: A Randomized Comparison With the Anterior Approach and a Cadaveric Evaluation | 2018 | Regional Anesthesia and Pain Medicine | Japan | UMIN000026748 |
| Zhai | Effects of a fixed low-dose ropivacaine with different volume and concentrations on interscalene brachial plexus block: a randomized controlled trial | 2016 | BMC Anesthesiology | China | ChiCTR-TRC-13004058 |
| Zhang | Combined ultrasound and nerve stimulator-guided deep nerve block may decrease the rate of local anesthetics systemic toxicity: a randomized clinical trial | 2019 | BMC Anesthesiology | China | ChiCTR-IOR-16008099 |
| Zhang | Comparison Between Subparaneural Upper Trunk and Conventional Interscalene Blocks for Arthroscopic Shoulder Surgery: A Randomized Noninferiority Trial | 2022 | Anesthesia & Analgesia | China | ChiCTR2000031178 |
| Zhao | Comparison of the anesthesia effect of ultrasound-guided middle and low interscalene brachial plexus block: a randomized, controlled, non-inferiority trial | 2023 | BMC Anesthesiology | China | ChiCTR2100054196 |
| Zhou | Single injection technique with ultrasound-guided superficial cervical fascia block combined with brachial plexus block in clavicular surgery: a prospective randomized comparative trial | 2023 | BMC Anesthesiology | China | ChiCTR2200064642 |
| Zhu | The ultrasound-guided selective nerve block in the upper arm: an approach of retaining the motor function in elbow | 2018 | BMC Anesthesiology | China | ChiCTR-IOR-16008769 |
| Zhuo | Ultrasound-Guided Clavipectoral Fascial Plane Block With Intermediate Cervical Plexus Block for Midshaft Clavicular Surgery: A Prospective Randomized Controlled Trial | 2022 | Anesthesia & Analgesia | China | ChiCTR2000038423 |
